# The Design Rationale and Preliminary Evaluation of a Prototype Designed by People With Lived Experience of Psychosis and Professionals: Design Research Study

**DOI:** 10.2196/80184

**Published:** 2025-12-04

**Authors:** Lars Veldmeijer, Gijs Terlouw, Job Van 'T Veer, Jim Van Os, Nynke Boonstra

**Affiliations:** 1 Research Group Health and Wellbeing NHL Stenden University of Applied Sciences Leeuwarden The Netherlands; 2 Kien VIP Mental Health Care Services Leeuwarden The Netherlands; 3 Department of Psychiatry University Medical Center Utrecht Utrecht The Netherlands

**Keywords:** co-design, psychosis, design research, dsign rationale, coproduction.

## Abstract

**Background:**

Experiences of mental distress are considered difficult to communicate, particularly experiences of psychosis. Research indicates that the frequently used medical focus falls short in capturing the nuanced interpersonal dynamics that these altered states may involve. Psychosis may seem very different from a lived experience perspective than it does from a traditional psychiatric perspective. This calls for innovative lived experience–based methodologies. This paper presents the development and preliminary evaluation of a design prototype co-designed to strengthen the role of people experiencing psychosis in the care process and describes its design rationale.

**Objective:**

The aim of this research is 2-fold. First, this study aims to co-design, in partnership with people with lived experience, an approach supported by generative design methodology that enables clients to express their experiences in their own way and evaluate the developed approach with dyads of clients who experienced a first episode of psychosis and professionals. Second, it aims to provide a clear and transparent design rationale for the approach, enabling future designers and researchers to understand its intention.

**Methods:**

The study involved co-design workshops, prototype sessions, a small-scale test phase with 7 client-professional dyads, the generation of qualitative data through semistructured interviews, and deductive and inductive thematic analyses.

**Results:**

The *In Picture Approach* was co-designed and tested, and its design rationale was described. The preliminary evaluation indicates that the developed prototype stimulated motivation, dialogue, and reflection of clients, with professionals reporting improved insight into their clients and some reconsiderations in care plans. While the exercises themselves were not always the source of new insights, the conversations they provoked proved meaningful. Clients felt seen and empowered and felt more able to take the initiative. Guided by design frameworks, the design process and design rationale were described, including design principles, supporting theories, working theory, design choices, and coproduced goals, allowing future researchers and designers to build on the concept.

**Conclusions:**

This study presents a lived experience–based, co-designed prototype that can be positioned as a potential boundary object, along with its design rationale. Preliminary test results suggest that *In Picture Approach* can foster meaningful dialogue between client-professional dyads, support clients’ self-exploration, and provide professionals with new perspectives on their clients. Although tested on a small scale, the results suggest its potential as a supportive tool within recovery-oriented care; however, broader and longer-term evaluation will be required to establish its contribution to personalized care planning. The co-design approach stimulated lived experience leadership by giving real decision-making power to people with lived experience of psychosis. Most importantly, this paper shows why it matters to make design rationales explicit in a field where they are often missing.

## Introduction

### Background

Experiences of mental distress are considered difficult to communicate. Contrary to the ontological assumptions embedded in, for example, *Diagnostic and Statistical Manual of Mental Disorders* classifications that implicitly localize these experiences within cerebral activity, experiences of mental distress are also bodily, sensorily, and socially embedded [1], making them contextual and personal. As a result, they are not always easily communicated, and prevailing approaches may leave insufficient space for clients to ascribe meaning to their experiences within standard psychiatric practice [2]. While psychiatric methods are primarily designed to assist professionals in diagnosis and treatment, a phenomenological and epistemic perspective emphasizes the importance of enabling patients to convey their experiences authentically, positioning them as legitimate epistemic agents during their care journey [3-5].

For those experiencing mental distress, exploring and expressing their experiences in ways that reflect their own meanings are important. Take psychosis, for example: research shows that the medical focus commonly used fails to capture the complex personal dynamics these altered states can entail [6]. From a lived experience perspective, psychosis may appear vastly different than how it is traditionally conceptualized in psychiatry [7]. Many people with lived experience regard hearing voices as a meaningful human phenomenon, one that is intelligible within the context of their life history [8]. A recent systematic review on subjective experiences indicates that delusions should not be reduced to dysfunctional beliefs, as they are complex, meaningful, and often coherent to the individuals who experience them [9]; such altered perceptions and extreme states can help people make sense of their lives and, in some cases, support their goals [10].

### Generative Design Research and Psychosis

Advancing our understanding of psychosis requires bridging research paradigms in mental health [11] by drawing on methods beyond its disciplinary boundaries. One promising paradigm for developing human-centered approaches to psychosis is design research. Generative design research typically starts with assessing needs and analyzing contexts of potential end users and treats individuals as experts of their own experiences. The methodology also offers diverse methods that empower them to explore and articulate those experiences within context [12]. Although initially intended for informing product and service design, these methods may be promising for experience-based and self-reflective research activities in mental health care, given that lived experience encompasses knowledge acquired through personal experience [2,13,14].

Evidence shows the field of design is receiving increased attention in the realm of psychosis research and practice [15]. These studies demonstrate that design approaches can validate experience and develop a shared language between professionals and service users, promoting a mutual understanding that extends beyond traditional frameworks [4]. Especially when drawing on boundary objects theory, prototypes can be designed to traverse perspectives without reducing them, establishing shared models and language across stakeholders [16]. However, the “design for health” field continues to struggle with explicating the rationale and foundations of these innovations [17-19], which are deemed essential for future designs and the evaluation of prototypes [20]. Design rationales are commonly defined as:

[...] important tools because they can include not only the reasons behind a design decision but also the justification for it, the other alternatives considered, the tradeoffs evaluated, and the argumentation that led to the decision. [21]

### Objective

The aim of this design study is 2-fold. First, this research aims to co-design an approach supported by a generative design methodology that provides clients with tools that enable them to share their personal experiences in a way that suits them best. Subsequently, we will evaluate this approach with dyads of clients who have experienced a first episode of psychosis and professionals. The hypothesis is that these tools can facilitate mutual understanding and inform more personalized care plans [22]. Second, it aims to describe a clear and transparent design rationale for the approach, enabling future designers and researchers to understand its intent and rationale by offering an example of how researchers can formulate and describe their design choices in research. The 2 objectives build on each other. The first focuses on creating and testing the approach, while the second justifies the choices behind it.

## Methods

### Study Design

**Figure 1:**
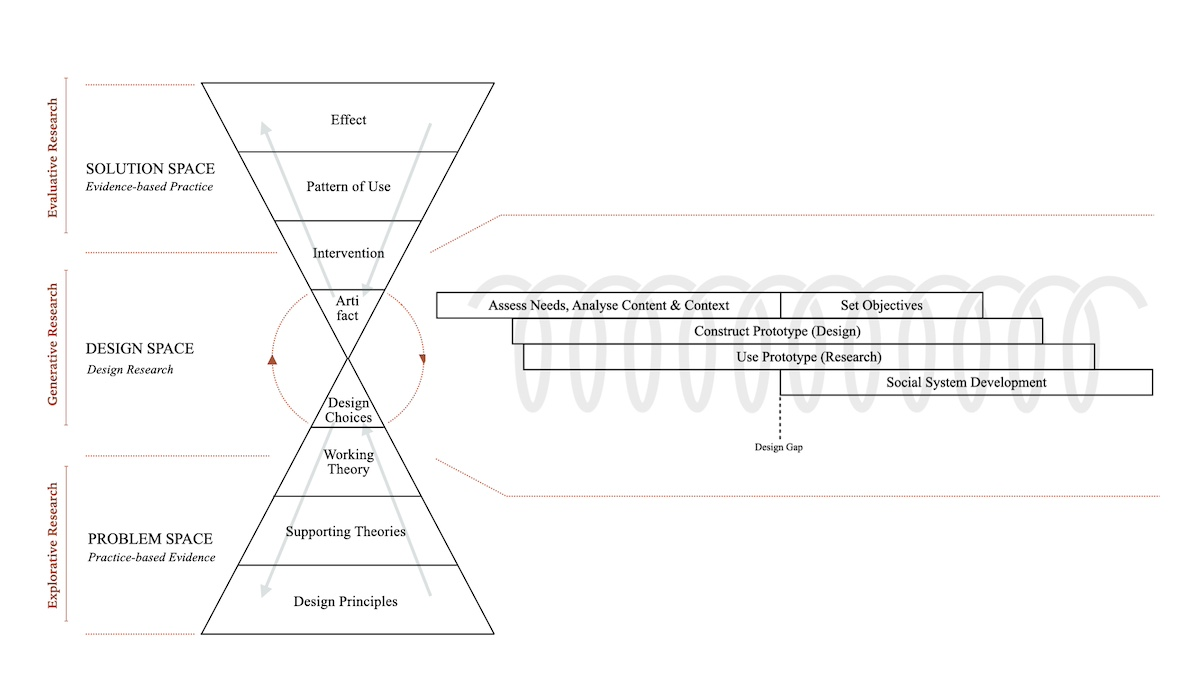
The Layers in Serious Media Design and Design Research Framework. Adapted from Kuipers et al [18]. The rights of the original Figure 1 are owned by a third party. The original figure has been adapted (the addition of Explorative Research, Generative Research, and Evaluative Research, all on the left of the model) but the meaning of the content has not been altered.

This study follows the Design Research Framework (DRF; Figure 1, the model on the right), which guides the creation of prototypes in health care and is a process that is both gradual and cyclical [18,23]. As the process unfolds, the emphasis of each cycle shifts, reflecting the nonlinear nature of design [24]. The DRF provides guidance throughout the design process, helping to clarify which aspects of the tool are being given attention during development. The study was conducted from 2023 to 2025 (Table 1).

**Table 1:** Timeline design and evaluation activities.

When	What
September 2023	Co-design workshops: assess needs and analyze context
December 2023 to February 2024	Expert sessions: construct prototype
March 2024	Co-design workshop: set objectives and social system development
March to June 2025	Preliminary evaluation: use prototype

Kuipers [20] describes the Layers in Serious Media Design (LiSMD) model (the hourglass-shaped model shown to the left of the DRF in Figure 1) as a cross-disciplinary framework that identifies key areas for developing innovative, purpose-driven artifacts. The DRF primarily describes the interplay between the problem space and the design space, in which choices are made explicit and tested through (design) hypotheses, searching for early indicators of success. According to Kuipers [20], this process produces a blueprint that not only encompasses the essential elements of the innovation but also creates testable prototypes, which can establish relevance and societal acceptance before entering the solution space. This study focuses on the problem and design spaces, aiming to assess usability and utility by identifying early indicators of success rather than definitive outcomes. During the design phase, informal feedback was collected after each workshop. The study was conducted within a mental health care organization in the Netherlands specializing in Early Innovative Psychosis Care.

### Person With Lived Experience Partnership

The level of involvement of clients and individuals with lived experience is usually low in mental health care research. A systematic review of design in mental health care shows that co-design and participatory design methodologies hold the potential for the highest levels of involvement [25]. To ensure that people with lived experience informed the scope of the study, the research team partnered with an individual who had lived experience of psychosis, contributing throughout the whole study design and codetermining how design sessions and research activities were shaped. This partner, who wishes to remain anonymous, ensured that lived experience continuously informed the design and research processes. This partner was involved in each stage of the research process, although their role varied throughout the various phases of the project. The partners (lead researcher and person with lived experience) met several times within every phase. We used the involvement matrix in partnership to describe this collaboration [26] (Table 2).

**Table 2:** The involvement of people with lived experience (as advised by Smits and colleagues [26]).

Project phase	Role in project	Actions
Preparation (study design and recruitment)	Advisor	Gave solicited and unsolicited advice on how to design the study and who to involve, proposed ideas for the design workshops and the interviews, and checked how their ideas were implemented in the study design.
Execution (design workshops and prototype design)	Decision maker	Took decisions on how the workshops were conducted, made together with the lead researcher the final decisions on the design principles and design choices, and made the final decisions on the visualization of final prototype.
Implementation (preliminary evaluation)	Partner	Worked as an equal partner alongside the research team in the prototype testing phase. Provided topics and gave feedback on the design and execution of the manuscript.

### Participants

Participants included individuals with psychosis, mental health professionals, and people with lived experience. Inclusion criteria for individuals with psychosis were (1) current or prior treatment within mental health services, (2) a diagnosis related to psychosis, (3) not in a crisis, (4) aged 18 years or older, and (5) legal competence to provide informed consent.

**Figure 2:**
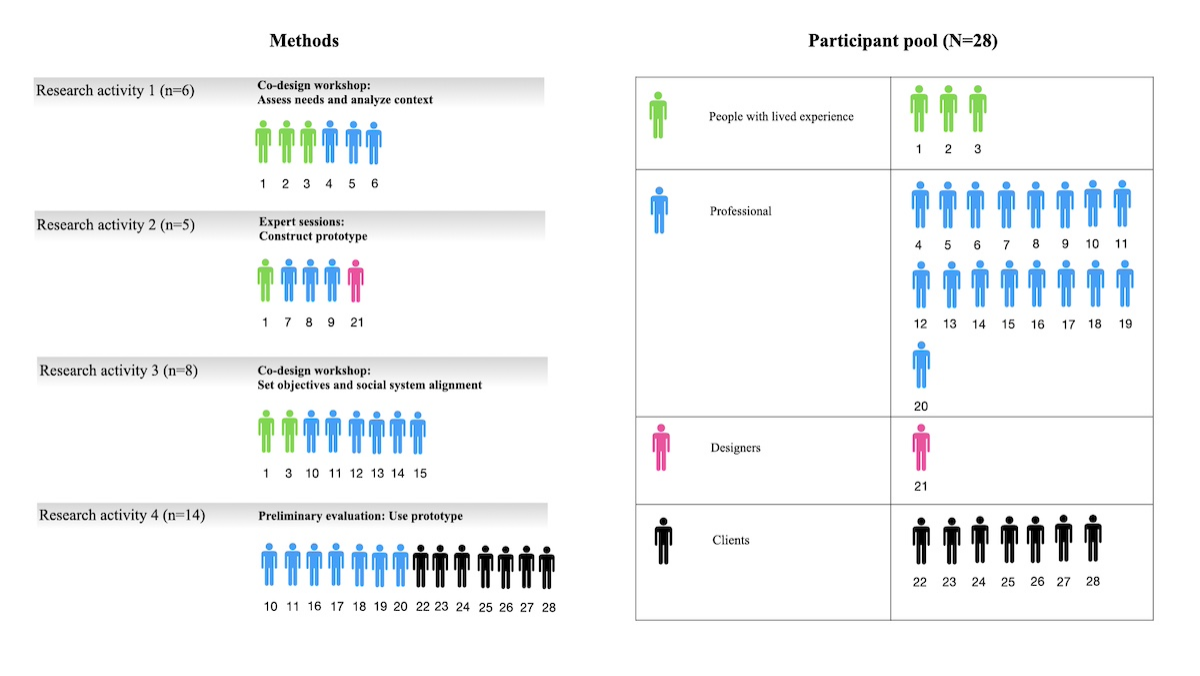
Participants per research activity.

Professionals were required to have (1) formal training in mental health care and (2) a current therapeutic relationship with one of the participating clients. The existing relationships were considered necessary, as advised by the lived experience partner, to safeguard trust in a sensitive psychiatric context. Each professional-client pair participated as a dyad. The goal was to recruit 8 such dyads, taking into account possible attrition. Besides having lived experience of psychosis, no other fixed inclusion criteria applied to people with lived experience. By “people with lived experience,” we refer to individuals who have experienced psychosis and are not currently receiving mental health services but have in the past. Upon completion, all participants received a €20 gift card (equivalent to US $23.27; Figure 2).

### Recruitment

Mental health professionals were invited to participate after a presentation of the study was held within the Early Innovative Psychosis Care organization. Clients were recruited through professionals with whom they had an existing professional relationship. These professionals, affiliated with the organization, assessed the suitability of the clients and introduced the study to them using the patient information folder. A research nurse coordinated recruitment and provided additional information. Upon confirmation, clients received a detailed information package and contact details for an independent psychiatrist who could be consulted. The client then formed a dyad with the professional. The independent psychiatrist also served as the neutral contact for complaints or conflicts, and this information was provided in the invitation letter that participants received prior to participation.

### Ethical Considerations

This study does not fall under the scope of the Dutch Medical Research Involving Human Subjects Act (Wet Medisch-Wetenschappelijk Onderzoek). It therefore does not require approval from an accredited medical ethics committee in the Netherlands. However, in the UMC Utrecht, an independent quality check has been carried out to ensure compliance with legislation and regulations (regarding the informed consent procedure, data management, privacy aspects, and legal aspects). NHL Stenden University of Applied Sciences Ethical Board also conducted an independent quality and ethical check and approved the study (reference 202502). All participants gave written informed consent. The research adheres to the Code of Conduct for Health Research (Gedragscode Gezondheidsonderzoek) [27], “Wet Geneeskundige Behandelingsovereenkomst” [28], Algemene Verordering Gegevensbescherming [29], and the EU General Data Protection Regulation [30].

### Co-Design Workshops: Assess Needs and Analyze Context

In September 2023, two 2-hour workshops explored how individuals give meaning to psychosis. The assignment was to co-design concepts in partnership that provide meaning to psychosis. Each workshop group consisted of at least 1 professional and 1 person with lived experience of psychosis. Master’s students in the Health Innovation program led the first workshop, with support and supervision from the first author (LV). The students received prior training in facilitation and ethical conduct in the part-time Master’s Health Innovation program and were already working professionals in health care who completed bachelor’s health care programs. In this workshop, generative techniques were used to enable participants to externalize the hard-to-verbalize aspects of their lived experiences through expressive methods, such as visual mapping, collage, and storytelling [12]. This workshop focused on concept development, while the second workshop, led by the first author (LV), evaluated the concepts using a Venn diagram and defined design principles. A total of 6 participants contributed (3 people with lived experience and 3 professionals, comprising a total of 3 groups). The professionals’ backgrounds were an arts therapist, a nurse practitioner, and a nurse (see Table 3 for the complete setup and procedural steps).

**Table 3:** Workshops setup and procedural steps.

Step		Duration	Activity	Purpose
**Workshop 1**				
	1. Introduction and framing	15 minutes	Welcome, explain aim, set ground rules	Establish a safe and collaborative environment
	2. Brainstorming needs and challenges	30 minutes	Storytelling, collages, visual mapping, and cluster into themes	Surface key issues and perspectives
	3. Concept development	60 minutes	Mixed groups developed concepts with low-fidelity prototyping	Translate perspectives into visual products
	4. Closing	15 minutes	Reflect on session, explain follow-up workshop	Prepare participants for workshop 2
**Workshop 2**				
	1. Introduction	15 minutes	Reflect on previous workshop	Look back on the first workshop, talk through the second workshop
	2. Sharing	30 minutes	Present low-fidelity prototypes, discuss and provide feedback	Encourage peer-learning
	3. Synthesis of shared goals	15 minutes	Visualized shared aims of the low-fidelity prototypes using a Venn diagram	Identify common objectives across concepts
	4. Distilling design principles	30 minutes	Guided discussion that identified recurring choices, mechanisms and values, translated into 5 design principles	Capture rationale behind the design of the concepts
	5. Member check	15 minutes	Checked with the participants whether the principles were accurate	Create a culture of shared ownership
	6. Closing	15 minutes	Reflect on session, thank participants, ask for feedback on workshop setup, and discuss next steps	N/A^a^

^a^N/A: not applicable.

### Expert Sessions: Construct Prototype

Between December 2023 and February 2024, 3 expert sessions were held to co-design a prototype. A total of 5 participants contributed (3 professionals, 1 person with lived experience, and 1 designer). The professionals’ backgrounds were a nurse practitioner, a clinical psychologist, and a psychiatrist. The designer also had lived experience. The prototype was iteratively shaped and visualized by the lead researcher, partner with lived experience, and designer with lived experience. Feedback from the expert group was collected on paper and incorporated at each step. The design rationale, including the design principles, supporting theories, working theory, and design choices, was documented using the LiSMD model and the DRF.

### Co-design Workshop: Set Objectives and Social System Alignment

In March 2024, professionals and people with lived experience participated in a follow-up workshop to determine how the prototype could be integrated into primary care. The Innovation Canvas was used in this workshop. A total of 8 participants contributed (2 people with lived experience and 6 professionals). The professionals’ backgrounds were a nurse practitioner, a systems therapist, a psychologist, an arts therapist, and 2 nurses. The participants co-designed procedures for introducing the tool to clients and discussed how its content would be used in practice. These insights helped shape the coproduced evaluation goals used later in the study.

### Preliminary Evaluation: Use Prototype

Between March and June 2025, seven dyadic interviews were conducted to evaluate the prototype and to capture how it shaped the shared encounter. Client-professional dyads received the prototype, a Polaroid camera with 10 instant films, and creative materials. A total of 14 participants took part (7 clients and 7 professionals). The professionals’ backgrounds were a nurse practitioner, an arts therapist, a systems therapist, and 4 nurses. Clients could freely use the tool at home; there were no mandatory activities, and the prototype was explicitly presented as nondiagnostic. Two weeks later, clients brought the prototype to a semistructured interview and were interviewed in dyads on their experience with the prototype. Each dyadic interview was scheduled for 1 hour. To encourage openness during the interviews, the interviewer (LV) emphasized the voluntary sharing of information, allowed pauses, and checked individually whether participants felt comfortable continuing. Clients kept the prototype after the interview.

### Analysis

Interview data from the “Use Prototype” phase were analyzed using both deductive and inductive thematic analyses [31], structured around predefined clusters derived from the coproduced goals. From these clusters, overlapping themes emerged. Two independent raters (LV and GT) coded the data, compared their coding, and reached consensus during the inductive phase through discussion. A third researcher checked the final themes (NB). A summary of the preliminary themes was shared with 2 dyads for member checking. Transcripts were coded in Atlas.ti (version 22.0.2; ATLAS.ti Scientific Software Development Gmb H). Recordings and anonymized data were securely stored on the SURF research drive of NHL Stenden University of Applied Sciences.

## Results

### Co-Design Workshops: Assess Needs and Analyze Content and Context

In the first co-design workshop, each design group moved over the course of 2 hours from brainstorming to concept development, ultimately creating a first concept, which was presented a week later. Three distinct concepts emerged. The first concept was a digital application that allowed users to log their personal experiences throughout the day. The second concept, a “talking board,” featured a board with 2 large white sheets to facilitate joint reflection by physically positioning the board between participants, thereby aiming to structure and support conversations about psychosis and coconstructing person-centered language. The third concept was a music-based tool in which clients curated playlists of meaningful songs that could be shared and discussed with professionals in therapeutic settings. For professionals, these tools provided visual and accessible ways to understand complex inner and contextual experiences. For participants with lived experience, the tools motivated introspection and empowered them to recognize and express their strengths.

**Figure 3:**
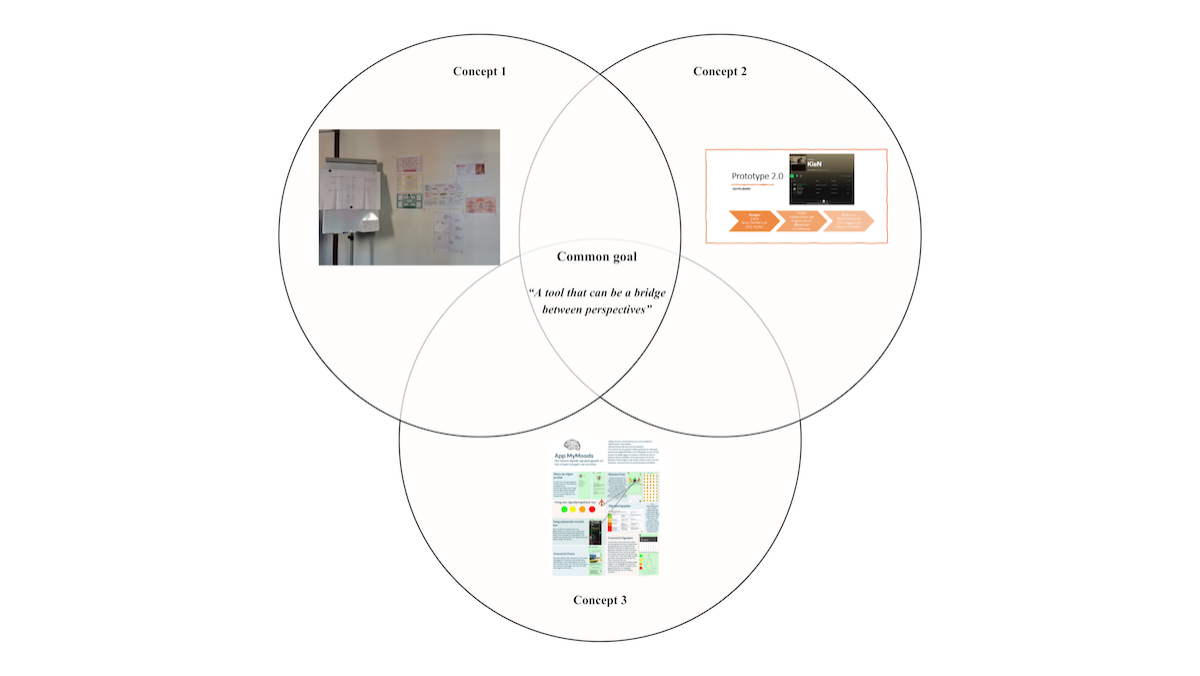
A Venn diagram showing the shared goal between the different concepts.

In the second co-design workshop, the aim was to uncover the rationale behind the prototypes. During this workshop, the participants analyzed shared design principles, the underlying mechanisms, and recurring choices. The evaluation aimed to understand not only what was created but also why and how form followed function in each case. Notably, none of the resulting design principles directly focused on interpreting psychosis itself. One person with lived experience explained, “In treatment, the main focus is already on psychosis; sometimes you want to talk about other things in your life. You are more than your psychosis,” while another added, “[...] and precisely because of that, by addressing those other dimensions, you learn to understand psychosis better.” The shared aim of the 3 concepts, as one individual with lived experience of psychosis noted, was “to build a bridge between the client and the professional,” a sentiment echoed by the other participants, visualized in a Venn diagram. A Venn diagram is a visual tool used to illustrate the relationships between different groups, concepts, or data sets, typically by using overlapping circles (Figure 3).

Under the guidance of the workshop coordinator (the lead researcher, LV), participants’ insights could be distilled into 5 design principles for the next iteration of a prototype that bridges perspectives:

#### Design Principle 1: Playful and Professional

The prototype should be both playful and visually appealing, while maintaining a professional quality that affirms the lived experience of clients and reinforces the professional identity of professionals.

#### Design Principle 2: Broad Topics

The content should remain general and positive to encourage spontaneous, intuitive interaction, avoiding overly clinical framing that might lead to overthinking hardships or reducing participants to their diagnosis.

#### Design Principle 3: Creative and Nonverbal

Rather than eliciting merely short verbal answers, the prototype should invite a wide range of nonverbal expressions and elicit intrinsic motivation through its nonmandatory form and content, thereby stimulating its use in everyday life.

#### Design Principle 4: Closedness and Openness

To be practical for professionals, the prototype must include clear and generalizable topics and yet remain flexible enough for clients to adapt it to their individual needs and lived realities.

#### Design Principle 5: Facilitating Future Perspectives

The artifact should move beyond symptomatology by highlighting strengths and encouraging users to envision positive futures, as creative and forward-looking approaches were found to be particularly motivating and generally lacking in contemporary psychosis care.

### Co-Design Workshop: Construct Prototype

#### The Design Process

During 3 expert group workshops, the various concepts from the co-design workshop were integrated into a single prototype based on the formulated design principles. Early on, participants envisioned a tool that clients could use independently in their living situations, allowing for personal expression on the one hand while generating contextual data to help professionals provide tailored support on the other. It was also the aim that the prototype would bridge the different perspectives involved. In the first expert session, the idea emerged to create a booklet with exercises that people could take home, which led to the initial concept called *Your Experience Book*. After receiving the feedback, it became apparent that the term “book” was controversial. “For some people, especially those who are less verbally inclined, this might evoke some resistance, possibly even associations with homework.” Therefore, the concept evolved into the *In Picture Approach* (IPA)*.*

Through developing the approach in different iterations, many adjustments were made. Some original exercises risked causing participants to focus too much on mental states or potentially triggering sensitive situations. Other exercises required more clarity in the instructions. A person with lived experience raised concerns that taking photographs might evoke shame, although it was also recognized by the same participant as a creative exercise with real potential to make experiences tangible. The final prototype addressed these issues by clearly and transparently explaining the use of photographs. For the sake of clarity, we will focus primarily on this final prototype version, as it is the one ultimately tested and evaluated, and the second objective of the study is to elaborate on the design rationale of the prototype.

Guided by the design principles, the expert group formulated themes drawn from their experiences and the results of the co-design workshop concepts to shape the booklet’s exercises. To preserve the democratic co-design approach, all experts (referring to all participants who contributed to the conceptualization of IPA during the “Construct Prototype” phase) were given equal decision-making power, and all exercises suggested by experts were included, allowing testing in practice to reveal which ones are most meaningful rather than selecting based on preliminary assumptions or prior research. The experts agreed that the prototype should be a physical booklet and not a digital app because professionals found apps complicated to use, and people with lived experience stressed that a tangible object could increase motivation to engage. The themes included (1) making unique experiences tangible, (2) personal biography, (3) music, (4) hobbies, (5) emotions, (6) social network, and (7) daily life.

#### The Artifact

**Figure 4:**
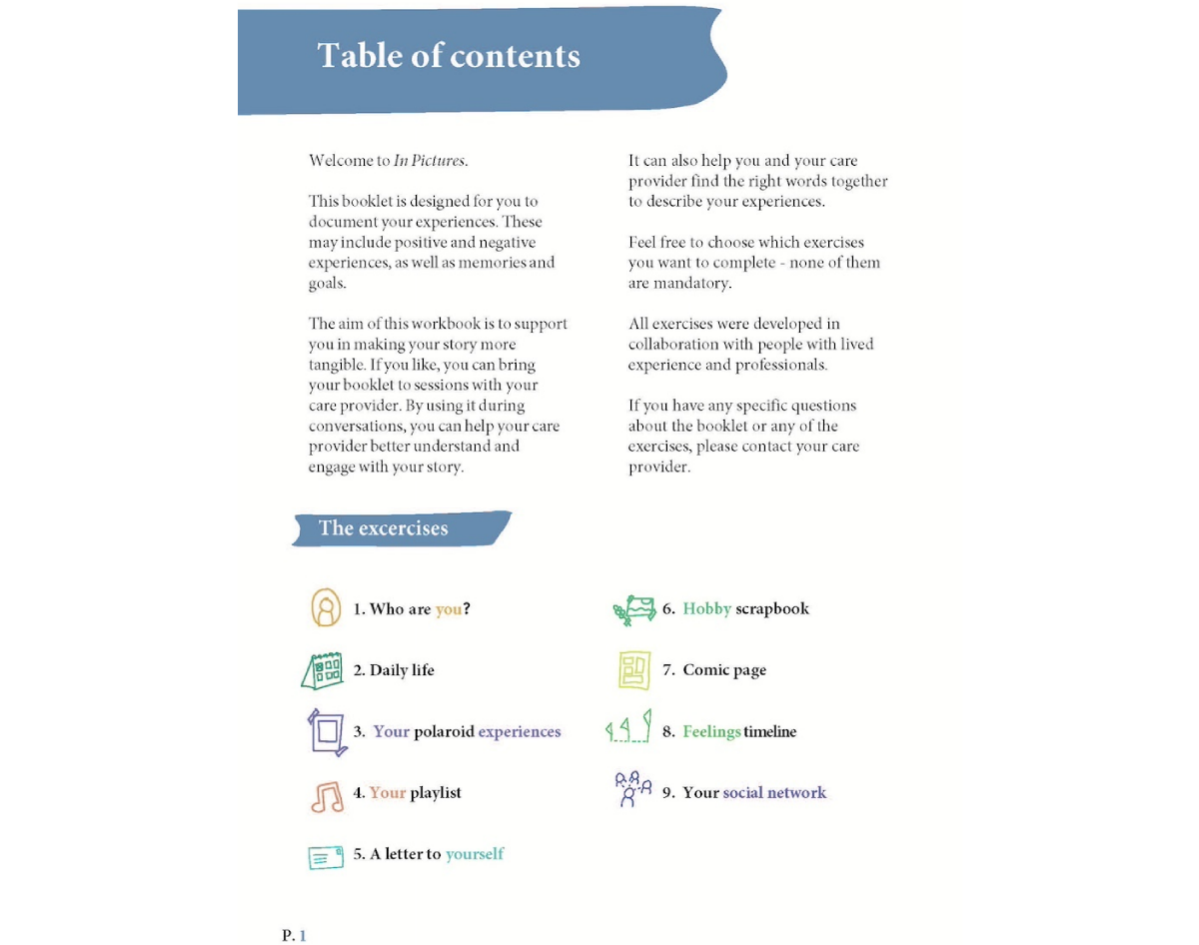
In Picture Approach table of contents (translated from Dutch).

**Figure 5:**
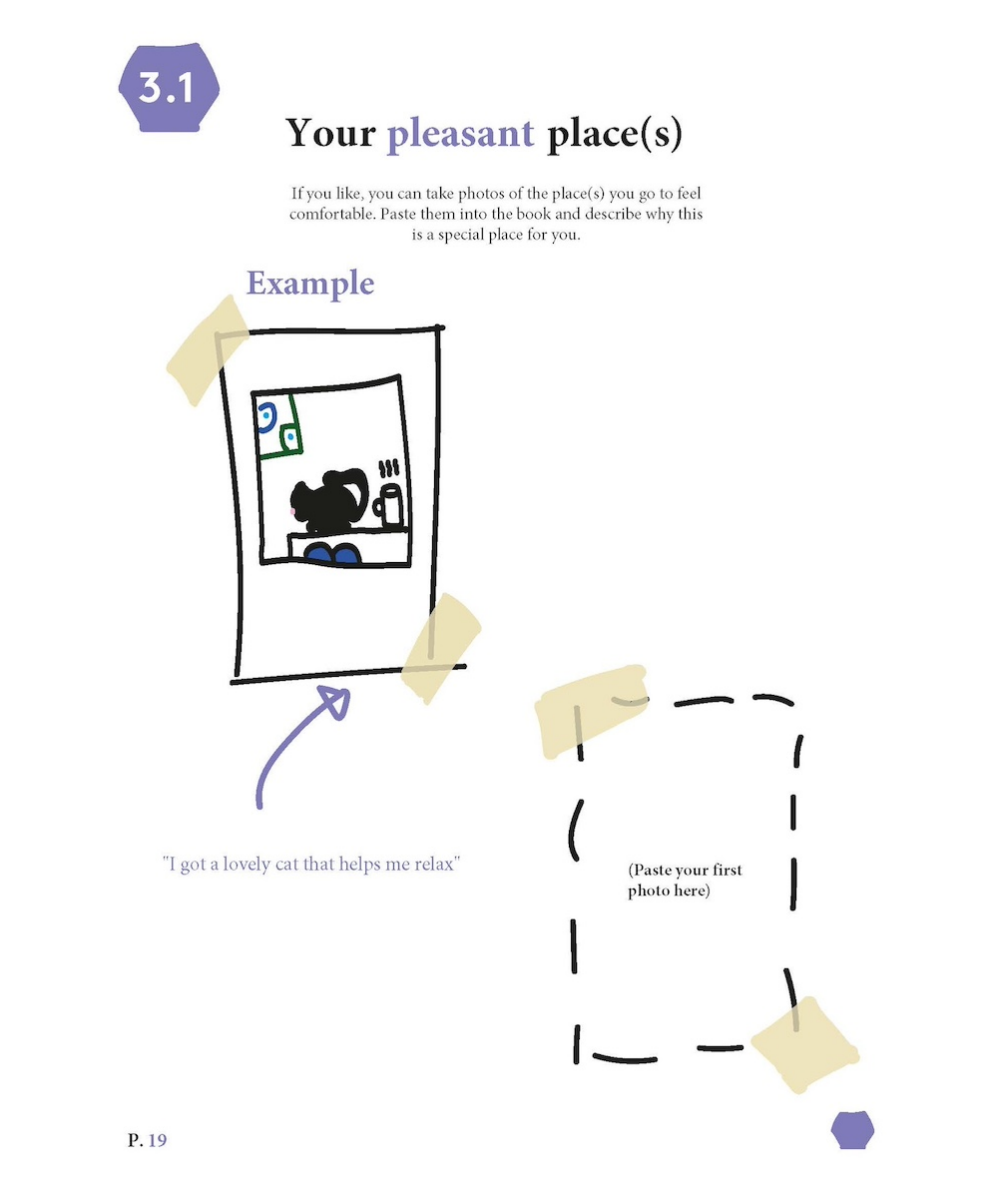
Example page ‘Your Pleasant Place (s).’.

**Figure 6:**
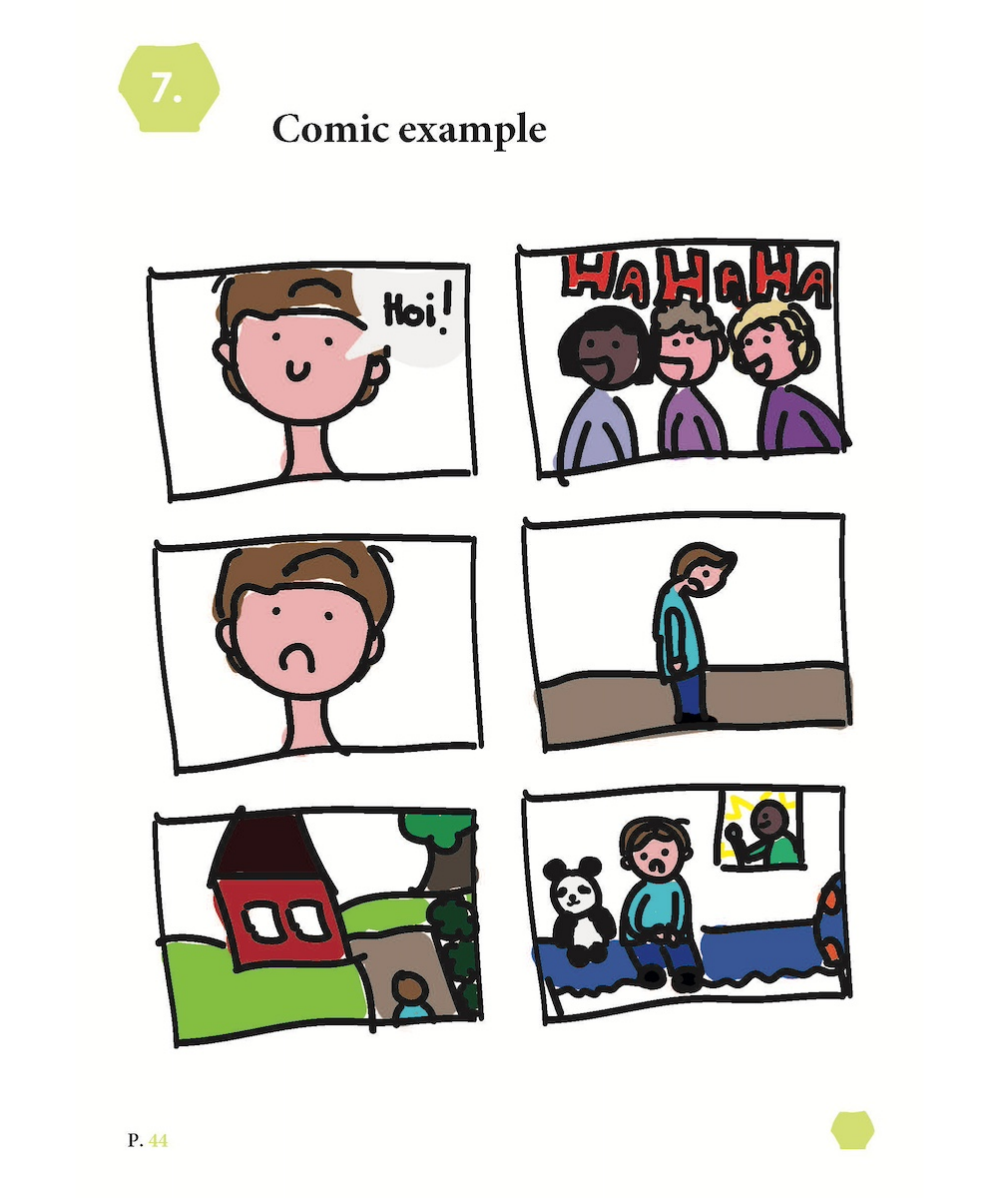
Example page "Comic Example.".

**Figure 7:**
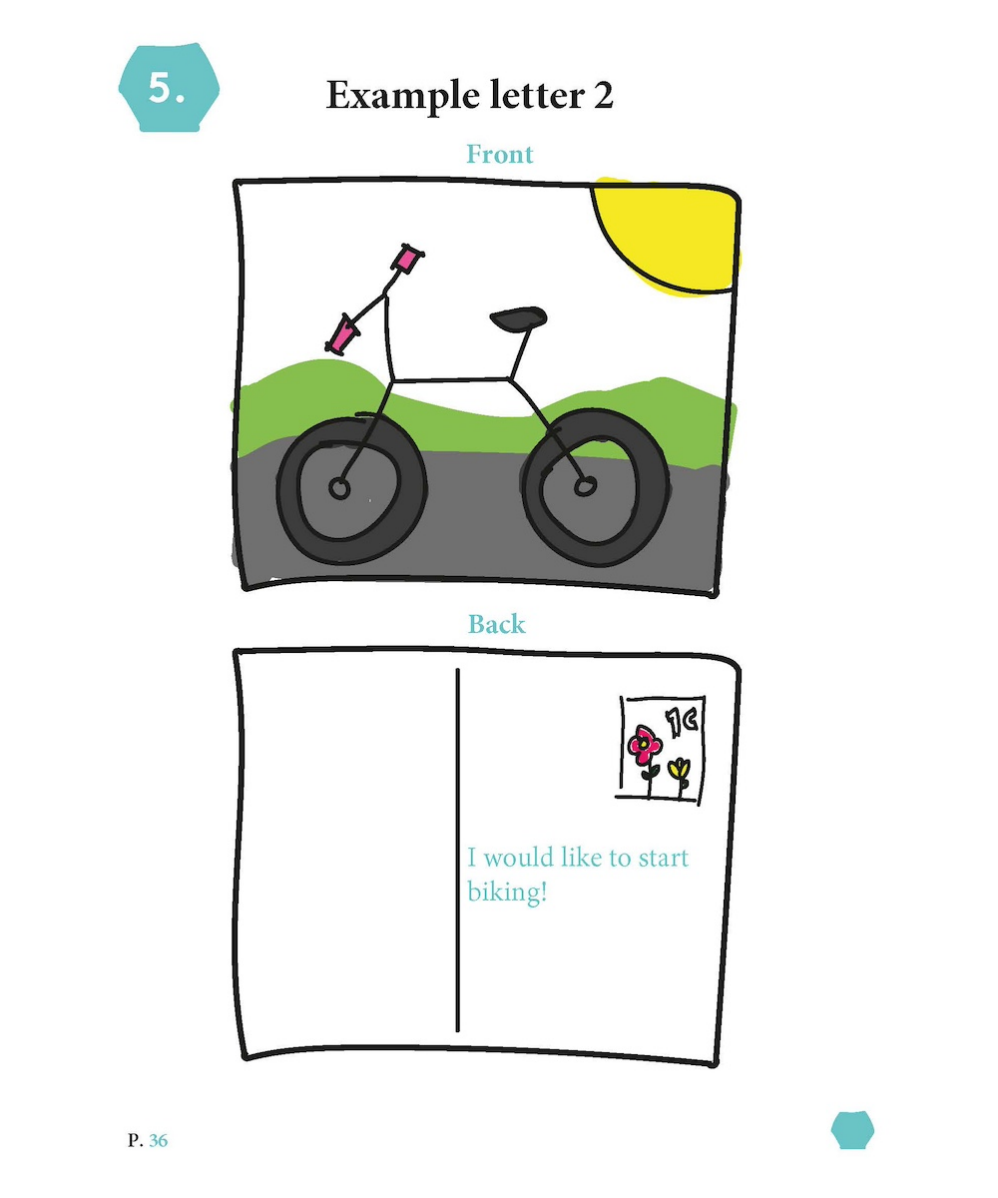
Example page "Letter to Yourself.".

The final artifact, named *In Picture Approach*, is a booklet containing exercises based on the 7 themes. None of the exercises in IPA are mandatory. The exercises, among others, include taking photographs of meaningful places, visualizing social systems, writing a letter to oneself, collecting personal artifacts, ranking meaningful songs, and creating a comic about one’s life (Figure 4; see Figure 5-7 for example pages). In Multimedia Appendix 1, we provided the complete booklet.

**Figure 8:**
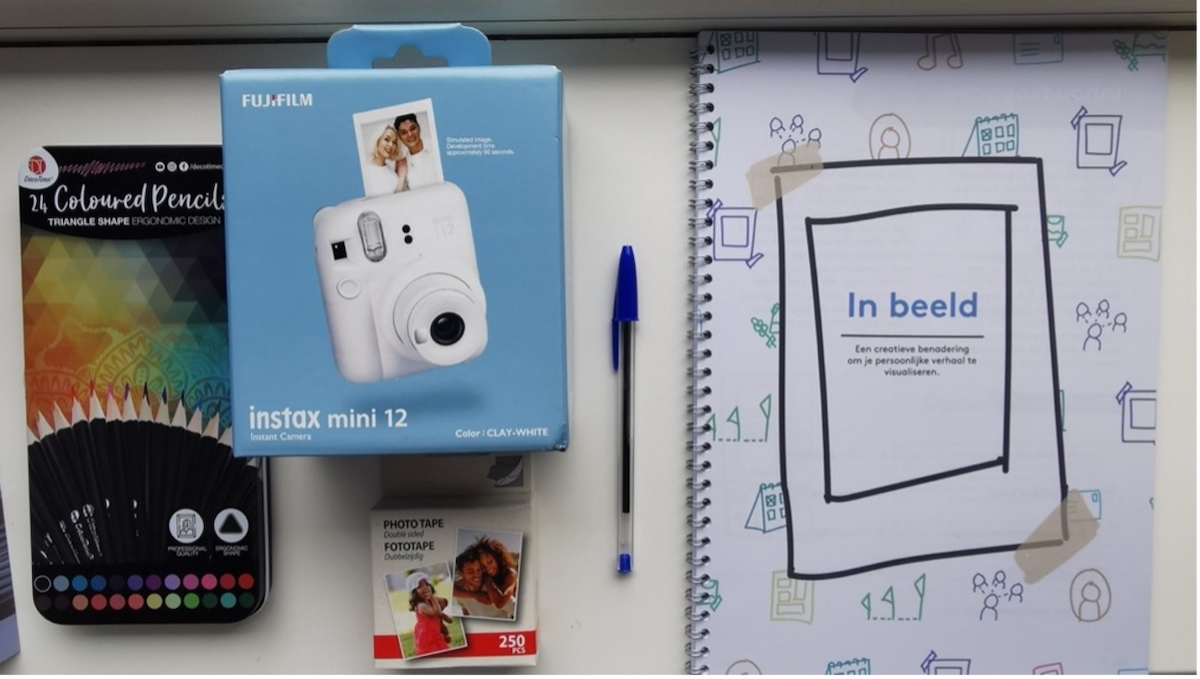
The prototype package (“In Beeld is Dutch for “In Picture”).

The exercises aim to help clients explore the themes mentioned before. The booklet is supplied with creative materials, including colored pencils and stickers, along with a Polaroid camera, instant film, and photo tape, to enable users to visually document their lives and add photographs directly to the booklet. The idea behind this was that IPA must feel like a gift rather than an assignment. Consistent with the design principles, the artifact strikes a balance between openness and structure, appealing to both clients and professionals, while focusing on future needs and avoiding pathological language (Figure 8).

The expert group concurred with the outcomes of the first 2 workshops (Assess Needs and Analyze Content and Context) and defined the goal of IPA as “building a bridge between client and professional perspectives in the therapeutic relationship while supporting clients in conveying knowledge about their mental and social experiences in their own way.”

#### Supporting Theories

To underpin the prototype with conceptual and practical insights [20], 4 theories were selected to support the design of the artifact. First, cultural probes, a qualitative research tool, provide users with tasks and materials to collect personal experiences within their own context, encouraging nonverbal self-reflection and the expression of needs [12,32,33], closely aligning with IPA’s concept. Second, boundary object theory describes objects that are interpreted differently by various social groups, but their structure is common enough to be recognizable across multiple worlds, serving as a means of translation [34]. IPA aims to function as such a potential boundary object by creating shared meaning without requiring agreement through perspective making and taking [16]. Third, Illich’s [35] philosophy of convivial tools inspired the artifact’s goal to empower clients to create something truly their own, allowing them to enrich their environment with their personal vision, which in our study, for example, refers to the democratization of the design process. Finally, we incorporated the concept of Personal Diagnosis [36], which reconceptualizes mental distress as human variation and advocates for an open “not-knowing” mindset that is based on curiosity. The four key areas of enquiry in Personal Diagnosis are as follows: (1) What happened to you? (2) What are your sensitivities and strengths? (3) What is important for you to strive toward? (4) What resources do you need to accomplish these goals? These questions, based on empirical evidence, closely overlap with the themes as formulated for IPA by the expert group.

#### Working Theory

The practical insights and supporting theories together form the working theory. A working theory is important to a design rationale because it constitutes a hypothesis that can be tested and used as a foundation for formulating goals. The working theory comprises the supporting theories and insights from the design workshop, captured in a single design hypothesis. It links the problem space with the design space (see the left side of Figure 1) and shows the contours of the artifact’s initial version [18]. The working theory for IPA is described as follows:

The artifact—IPA—enhances the ability of people with psychosis to express their own narratives in their own way. The exercises in the artifact produce rich, situated knowledge that evokes conversation, connection, and meaningful reflection. IPA, in turn, becomes the engine for joint decision-making and contributes to care that is built on mutual understanding and relational alignment by making personal perspectives and needs transferable and conversation-worthy within the professional context.

#### Design Choices

The design process and working theory led to several specific design choices. Following the LiSMD model, these choices were informed by the working theory and integrated both practical insights and theoretical frameworks [20]. When we refer to participants, we include both individuals with lived experience and professionals.

#### Using Themes Centered Around Identity, Everyday Life, and Preferred Futures

The exercises in IPA are based on themes such as personal biography, emotions, social network, and hobbies. These were chosen based on the requests of participants to allow clients to share their unique stories, including aspects of life often excluded from strict medical discourses, and on the theory of Personal Diagnosis [36]. In addition, pathologizing language was avoided, and no exercises were created that explicitly focused on the experience of psychosis, allowing space for broader themes that participants found important (design principle 2). Also, exercises were developed that instead of focusing merely on the past, concentrate on preferred futures (design principle 5).

#### Integration of Creative Materials and Photography

IPA includes a kit with creative materials (eg, colored pencils and stickers) and a Polaroid camera. Participants hypothesized that creative materials enable clients to visually and expressively capture aspects of their lives that may be difficult to articulate in words but can reveal a great deal about their circumstances (design principle 3). This design choice aligns with the supporting theories of cultural probing and context mapping, which also emphasize the collection of personal artifacts and nonverbal expressions [32,33].

#### Nonmandatory Nature of the Exercises

The exercises in the prototype are intentionally nonmandatory. This preserves client autonomy and respects their individual pace and preferences (design principle 3). The aim is not to standardize but to create space for subjective expression and meaning making [35]. In addition, the design intentionally avoids appearing too polished. Participants noted that an overly “finished” or stylized format may discourage participation by making clients feel their own creative abilities are inadequate.

#### Bridging Client and Professional Perspectives

The prototype is explicitly designed as a potential boundary object that facilitates communication between the client’s personal perspective and the professional framework (design principle 1). By gathering personal, contextual information through stories, images, and creative expression, professionals gain a deeper understanding of the client, while clients gain agency over their narrative (design principle 4). Boundary objects theory underpins this bridging function [34], which participants explicitly wished for. According to boundary objects theory, this can trigger the dialogical learning mechanism of reflection, which potentially supports perspective-taking and making [16].

### Co-Design Workshop: Set Objectives and Social System Alignment

Following the development of a prototype through co-design, it became possible to systematically identify stages within the care process where its implementation and evaluation may be appropriately situated according to people with lived experience and professionals. Therefore, in this section, we present the results of the Innovation Canvas workshop.

#### Opportunities That IPA Potentially Offers in the Care Process

Participants, including individuals with lived experience and professionals, expressed that IPA can provide several valuable opportunities throughout the care journey. According to the participants, this approach is the first of its kind within this mental health organization, as it empowers clients to control their own information, allowing them to contribute self-selected details and take the lead. This may facilitate what one professional called “problem-free conversations,” meaning discussions focused on life domains that do not typically arise in strictly problem-oriented or medical approaches. According to a person with lived experience, IPA may also help professionals understand clients more quickly, thoroughly, and from different perspectives compared with symptom-focused methods. As such, participants perceive IPA as an incremental improvement that allows professionals to see the person behind the symptoms without needing to steer the interaction, offering a new way of gathering information. Another benefit hypothesized by the professionals is increased treatment adherence due to the commitment IPA fosters, which aligns with the preferred phase of use: after diagnosis but before treatment begins. By enhancing the client’s role in planning, IPA may boost intrinsic motivation for treatment. In addition, people with lived experience hypothesized that the exercises potentially encourage reflection, making it easier for clients to express and clarify their needs.

#### Potential Risks of Implementing IPA in the Care Process

During the session, several potential risks related to IPA were visualized. One concern for a person with lived experience is that clients may struggle to complete the booklet quickly if IPA is introduced only at the end of the diagnostic phase, a period typically focused on finalizing diagnostics and initiating treatment. This “pressure cooker” conflicts with IPA’s nonmandatory nature, as clients need time to work through the exercises without feeling pressured by deadlines. While having sufficient time is essential, professionals often face time constraints. Professionals suggested that these risks could be mitigated by involving motivated clients for whom IPA is a good fit at that stage and assessing whether IPA suits the client’s personality and motivation. This requires proper instruction on its use. However, according to people with lived experience, even if a professional thinks IPA is not suitable, the client should have the opportunity to decide whether they find value in it. Another logistical risk is that clients may keep the materials for extended periods, which could prevent others from accessing them, as cameras are loaned out. This risk is directly connected to organizational preconditions that must be met to use IPA responsibly. Another risk concerns storage and privacy issues. People with lived experience, therefore, proposed that the booklet, both during and after usage, remains in the possession of the client, who would bring it to sessions and take it home afterward, ensuring that sensitive personal material stays under their control. Finally, all participants agreed that for conversations about the booklet’s content, a safe environment and an open, supportive attitude from the professional are essential so that clients feel free to share and photograph as they wish.

#### Coproduced Goals

Finally, the group discussed key goals for evaluating the prototype to test whether the working theory holds and to determine which goals the artifact should be assessed on. These goals were coproduced with people with lived experience and professionals and set during the workshop: (1) to motivate clients to engage in self-exploration, (2) to support clients in communicating their knowledge and needs, (3) to help professionals gain a deeper understanding of their clients, and (4) to contribute to the development of future care plans in collaboration with clients.

### Evaluation: Use Prototype

This section presents the results of interviews coded in Atlas.ti and analyzed using a deductive and inductive approach. First, we labeled the transcripts based on the coproduced goals, which were the predefined themes for deductive analysis (ie, Self-Exploration and Motivation, Communication of Needs, Professional Understanding of Clients, and Collaborative Care Planning), and second, we let overlapping themes emerge from these 4 clusters in our inductive analysis, which resulted in the final themes “Perspectives and Reflections” and “Care and Services.” In addition, we labeled every statement about the prototype, which also became a separate theme, namely, “The Prototype.”

#### Theme 1: Perspectives and Reflections

In most interviews, clients reported gaining a new perspective on themselves, while many professionals noted gaining new insights into their clients. Reflection was also stimulated; one professional shared that after evaluating the prototype with a client, they reconsidered their own current working methods. These insights primarily arose not from the exercises themselves but from conversations about their meaning and the exploration of various interpretations. According to the participants, it was the dialogue, rather than task content, that produced meaningful and helpful understanding. For some, the prototype mainly confirmed what they already knew, which they experienced as positive affirmation. One client described it as:

[...] a confirmation of what I already knew but could not easily express. [D4]

A few professionals with long-standing relationships with their clients found no surprising new insights. All clients felt motivated to engage in self-exploration, a perception confirmed by all professionals. Clients, often proudly, showed their (un)finished booklet, having chosen which exercises to complete. Although not all clients finished all exercises, all clients completed most of them. Several professionals described the results as “impressive.” Some client-professional dyads mentioned surprising discoveries related to the client’s strengths and needs.

#### Theme 2: Care and Services

In all interviews, professionals reported that the prototype helped them gain a better understanding of their clients. Professionals noted that personal contexts had become suddenly visible, providing insight into clients’ living situations and preferences. For most professionals, this deeper understanding primarily stemmed from the visual exercises, which provided an authentic portrayal of the person behind the symptoms and revealed unique qualities and personality traits. In a few client-professional dyads, the prototype motivated potential care plan adjustments, with new insights leading to discussions between dyads that concluded a change in direction that might better meet the client’s needs. The most motivating aspect for almost all dyads was the alternative, more positive approach to psychosis and self-concept. One client shared:

[...] having a positive perspective on who I am helped me regain trust in myself. [D2]

This was also recognized by their professional. Another client noted:

[...] I’ve spent many years in mental health care, but I’ve never been asked to explore what I’m good at or what my strengths are. [D7]

A few clients found the large number of exercises somewhat discouraging and sometimes confronting and reported feeling overwhelmed by receiving all the material at once. Despite this, all dyads agreed that the prototype could contribute to future treatment and recovery. However, a professional cautioned that this needs to be assessed later, once the prototype is used for a more extended period and with a larger group of clients. Clients were more optimistic; one said:

It is really a different approach: this time I got a gift [the prototype package] and was trusted with a camera, while in other institutions I was just sent questionnaires. [D5]

Several others remarked that they felt seen and heard as human beings. A small number of dyads felt that the prototype could support greater ownership allocated to clients. All dyads agreed that the prototype supports shared decision-making, offers insight into the care journey, and enhances communication between the client and professional.

#### Theme 3: The Prototype

According to almost all clients, the prototype’s principal practical value was its capacity to help them tell their own story and express what they wished to share. One client noted:

With IPA I bring something of myself to show [name professional], like ‘look, this is who I am. [D1]

Professionals acknowledged this shift; one said, for example:

[...] and that [IPA] allows me to ask more meaningful questions; it leads to a very different kind of conversation [compared to without IPA]. [D6]

Another value was its empowering effect. Most clients brought extra materials not requested by the exercises, and in a few cases, they also provided unsolicited feedback for the lead researcher, including one client who personally handed over a thank you letter with suggestions for new exercises.

The most common challenge was the limited timeframe for using the prototype. Some professionals found 2 weeks too short to work with IPA, especially if they wanted to do justice to the time and effort a client had put into the exercises. Another challenge was the lack of follow-up: more than half of the dyads raised questions about continuing its use. Another challenge was the nonexistence of a second edition. One client offered to help develop a second version. While most clients said that they were able to express their thoughts and feelings, some clients noted the absence of an exercise on psychosis. One client suggested including a page for so-called “extraordinary experiences.” According to the client, these exercises do not necessarily have to focus on psychosis but on experiences that have had a significant impact on who service users are as persons and how they ended up in care.

All client-professional dyads stated that they would recommend the prototype to others, although 2 dyads emphasized the importance of clients being prepared for it. A dyad stressed that if one has difficulty opening up or is not very creative, they might struggle, and then IPA could feel like a burden. Both clients and professionals proposed only minor changes and expressed general satisfaction with the concept. Suggestions included new exercises, such as a visual exercise to make extraordinary experiences tangible, improved wording, and refinements to form. For example, the future exercise was perceived as looking too far ahead; clients indicated a preference for focusing on the upcoming week rather than the next year, as they believed that this results in more achievable goals.

## Discussion

### Principal Results

The objectives of this study were to co-design an approach that provides clients with tools that enable them to share their personal experiences in a way that suits them best and to describe a clear and transparent design rationale for the developed approach. In this section, we will address both objectives.

First, the co-design process has delivered an approach, the so-called IPA, which we have subsequently tested in practice. Testing showed promising preliminary results. For example, all clients reported feeling motivated to engage in self-exploration, showing pride when presenting IPA and making selective use of the exercises. A few clients completed all exercises. It seems that clients felt free to engage with what they found meaningful. Most clients experienced the prototype as a way to share their stories, indicating that they were able to present themselves as human beings rather than merely clients. Clients often brought in personal materials or gave unsolicited feedback, including one suggestion to add an exercise on so-called extraordinary experiences. This can be interpreted as reflecting ownership over what they considered meaningful and relevant. The goal of self-exploration was unanimously recognized by clients, even when it did not always produce new insights. Furthermore, all professionals reported that the prototype helped them gain a better understanding of the client. Clients and professionals alike emphasized that the actual value of the prototype emerged through shared interpretation. Thus, according to this study, it appears that the meaning-making process of subjective experiences of mental distress and the personal dynamics of the client occur within relationships, where there is space for cocreative inquiries and person-centered experiments, such as IPA. Person-centered and cocreative approaches demonstrate the potential to function as relational mediators, potentially triggering the reflective dialogical learning mechanism [16]. Not all coproduced goals were equally evident across all cases. For example, only a few dyads reported a direct potential impact on the care plan, emphasizing that the current findings should be considered preliminary indications of the prototype’s potential and not definitive evidence of personalization. Nevertheless, the findings highlight the often underestimated epistemic legitimacy and collaborative capacity of people with psychosis within mental health contexts [2-4].

Second, we have described a clear design rationale for this co-design prototype. Thus, this paper demonstrates that designers, researchers, and people with lived experience, working collaboratively in the field of “design for health,” can give insight into a thoughtfully aligned design process from which informed design choices emerge, marked by what Kuipers [20] describes as “conceptual continuity” and what Hovingh and colleagues [19] refer to as an “underlying narrative that integrated the most important chosen components.” The presentation and explanation of those choices can inform future designers and researchers with a clear understanding of why IPA was developed, what its intended use was, how the artifact was tested in relation to the intended use, and how this all relates to the broader, generalizable effects that are considered necessary within the domain of health. These results are compatible with the definition of design rationales [21]. At the same time, models such as the DRF are not a quick fix to guarantee a sound design process; at best, they provide a useful framework for identifying key areas of focus within it.

### Design Research With People With Lived Experience

The IPA design process illustrates the importance of treating people with lived experience as authoritative agents who are “in a position to know” [25,37]. People with lived experience were involved throughout the whole research process and had decision-making authority [25]. The legitimate epistemic perspectives contributed by people with lived experience were decisive in ensuring that the developed prototype catered to the needs of the target group. However, during the design process, lived experience perspectives and needs were not always in line with those of professionals, resulting in a few meaningful collisions [38]. We managed this by incorporating both lived experience–based and professional-based exercises into the design of IPA and then letting the preliminary evaluation (testing) reveal what worked and what did not. The IPA process demonstrates that involving people with lived experience as architects of the design and research process can lead to user-led service approaches that are difficult to conceive without incorporating their expert perspectives. However, while the DRF was an appropriate method for articulating the design rationale of IPA, a different methodology, such as the experience-based co-design approach, could have also been suitable for integrating the participatory perspectives [39].

The result is a prototype shaped by the language and preferences of people with (experience of) psychosis, while at the same time aligning with the needs of the professionals. While the participation was uneven across phases, a person with lived experience (the partner) was a final decision maker in the design phase, which served as a meaningful and crucial counterweight to this asymmetry. This prototype was designed as a potential boundary object and, despite, or perhaps because of its varying types of usage, triggered meaningful conversations that bridged the perspectives of both parties in dyads [16]. This feature of boundary objects, which aims to connect the objectives of different stakeholders [40], can be very powerful to realign power balances in mental health care. Clients recognized themselves in the exercises and felt invited to contribute, while professionals were reinforced in their professional role, indicating that the identification mechanism was triggered [16]. Some clients added their own materials and produced their own “convivial tools” [35]. These tools can be interpreted as representational artifacts [41] that, in the case of IPA, promote an idiographic approach to experiences of mental distress [36]. These contributions, along with the interview results, suggest that clients experienced the prototype as an artifact of trust and recognition that has the potential to enable clients to flourish across mental health care services [42].

### Design Considerations and Framing of Psychosis

Future prototypes may explore several directions. For example, the use of IPA in group settings can potentially lead to peer-to-peer meaning-making and support through the sharing of narratives and creative contributions. Another direction that can be explored is making the client curators of their own personal version of IPA. Instead of providing only predeveloped exercises, IPA can also include open-ended templates and blank pages with gentle guidelines that enable clients to design their own reflective practices. These lived experience–based contributions, such as one client’s idea to create an exercise about extraordinary experiences, may also resonate with other clients, potentially giving rise to a growing, evolving collection of user-generated exercises. Such a database could be collaboratively managed by a team of people with lived experience and professionals to ensure that the developed activities remain both safe and meaningful for other clients.

Finally, we turn to the question of which lessons can be learned from the design process regarding how psychosis can be reconsidered or “framed” from the perspective of lived experience in design projects. Across the design workshops and interviews, people with lived experience and clients offered rich, in-depth first-person accounts of what psychosis means to them. Their descriptions portray psychosis as a shift in awareness, in which everyday experiences of life become hypermeaningful and charged with emotional, spiritual, and social significance. Some described it as tuning into a deeper layer of reality, where everything feels interconnected and deeply personal. From this lived experience perspective, psychosis can be framed as a profoundly human mode of meaning-making that is also overwhelming and disruptive. The resonance many clients expressed with IPA may therefore stem from its practical use, as it mirrors their experiences of psychosis as something to be explored and expressed. When participatory design centers first-person perspectives like these, it has the potential to create empathy and challenge and reframe dominant narratives of (mental) health and distress [43,44], which can benefit the development of novel approaches that are compatible with an era that requires human-centered approaches to mental distress [45], in particular to psychosis [15,46].

### Comparison With Previous Findings

This study is not the first design study carried out with people who have (experience of) psychosis. Previous research, for instance, with young people experiencing psychosis has shown that people with mental health conditions are well able to articulate their needs and that doing so opens up new directions for solutions [47]. Other studies have highlighted the added value of lived experience perspectives in developing a virtual reality intervention for people with psychosis [48], while others, much like the IPA study, emphasized that people with lived experience can be strong collaborators in a design process [49] and sometimes even touched upon the therapeutic potential of the activity of designing itself [50]. These findings overlap with the results in this study.

What distinguishes this study lies mainly in making the design rationale explicit. Whereas most studies focus on the preliminary or final outcomes of a prototype—which we have also addressed in this paper—the IPA study concentrates on what happens “under the hood”: which choices were made, how these choices relate to the literature, who was involved in the process, and on what principles the prototype was built. This design rationale creates a form of falsifiability. By taking the design rationale of IPA as an example, future design researchers can replicate the choices, examine the effects of similar approaches in different mental health care settings, and elaborate their own “blueprint” in greater detail: an aspect crucial for upcoming design projects in mental health care [15].

### Strengths and Limitations

A key strength of this study is the cocreative design research process, which used the DRF and LiSMD model, along with the Involvement Matrix, involving professionals and individuals with lived experience at every stage and reporting on their contributions in detail. Another strength is the rich qualitative dataset, generated through design workshops and semistructured dyadic interviews with client-professional dyads. Interviewing both sides of the care relationship simultaneously allowed for a deeper insight into the dynamics of mutual understanding [22] and usage of IPA in context. Deductive and inductive analyses of the semistructured dyadic interviews, along with methodological transparency, enhanced the reliability of the findings [31]. From a pragmatic design research perspective, another strength is the traceability of design choices [20,24] and their resonance in practice [51]. The outcomes are therefore not evidence of effectiveness in the traditional sense but they do provide an early indication of how the prototype performs in context and what kinds of value it generates according to potential end users.

Nevertheless, the study has a few limitations. First, due to privacy reasons, we were unable to share the creative work of the participating clients, which was very impressive. Second, the preliminary test period was short, there was no follow-up, the sample size was small, the clients were selected instead of randomly recruited, and the testing of IPA within existing relationships could have hindered the openness of the participating clients. Third, the professionals involved were already working from the principles of Personal Diagnosis. For those unfamiliar with this approach and relying more on traditional methods, working with approaches such as IPA may prove more challenging and require additional training to be successfully implemented in practice.

Finally, a limitation to acknowledge is the influence of the researcher-facilitator. The first author (LV) has lived experience of mental health care, which shaped both the facilitation of the process and the interpretation of findings. At the same time, other members of the research team contributed complementary perspectives from design, clinical practice, and academic research, which helped balance potential biases in the research team. However, the skills, personality, and specific interests of the first author inevitably informed the dynamics of the sessions and the emphasis in the analysis, which was a strength and a limitation at the same time. Thus, replication requires awareness of how a facilitator’s background and style can affect the process.

### Conclusions

This study presents a lived experience-based prototype called In Picture Approach, and its design rationale. Preliminary results suggest that IPA can foster meaningful dialogue between client-professional dyads, support clients’ self-exploration, and provide professionals with new perspectives on their clients. Although tested on a small scale, the results suggest its potential as a supportive tool within recovery-oriented care; however, broader and longer-term evaluation will be required to establish its contribution to personalized care planning. Most importantly, this paper shows why it matters to make design rationales explicit in a field where they are often missing, and it demonstrates lived experience leadership by giving real decision-making power to people with lived experience of psychosis.

## Appendix

Multimedia Appendix 1 The In Picture Approach Complete Booklet.

## Abbreviations

DRF: Design Research Framework
IPA: In Picture Approach
LiSMD: Layers in Serious Media Design
