# Supplementary material for: The Design Rationale and Preliminary Evaluation of a Prototype Designed by People With Lived Experience of Psychosis and Professionals: Design Research Study
Source: J Particip Med. 2025 Dec 4;17:e80184. doi: 10.2196/80184 (PMC12715466; doi:10.2196/80184)

In Picture

A creative approach to
visualizing your personal story.

Table of contents

Welcome to *In Pictures*.

This booklet is designed for you to document your experiences. These may include positive and negative experiences, as well as memories and goals.

The aim of this workbook is to support you in making your story more tangible. If you like, you can bring your booklet to sessions with your care provider. By using it during conversations, you can help your care provider better understand and engage with your story.

It can also help you and your care provider find the right words together to describe your experiences.

Feel free to choose which exercises you want to complete - none of them are mandatory.

All exercises were developed in collaboration with people with lived experience and professionals.

If you have any specific questions about the booklet or any of the exercises, please contact your care provider.

The excercises

1. Who are you?
2. Daily life
3. Your polaroid experiences
4. Your playlist
5. A letter to yourself
6. Hobby scrapbook
7. Comic page
8. Feelings timeline
9. Your social network

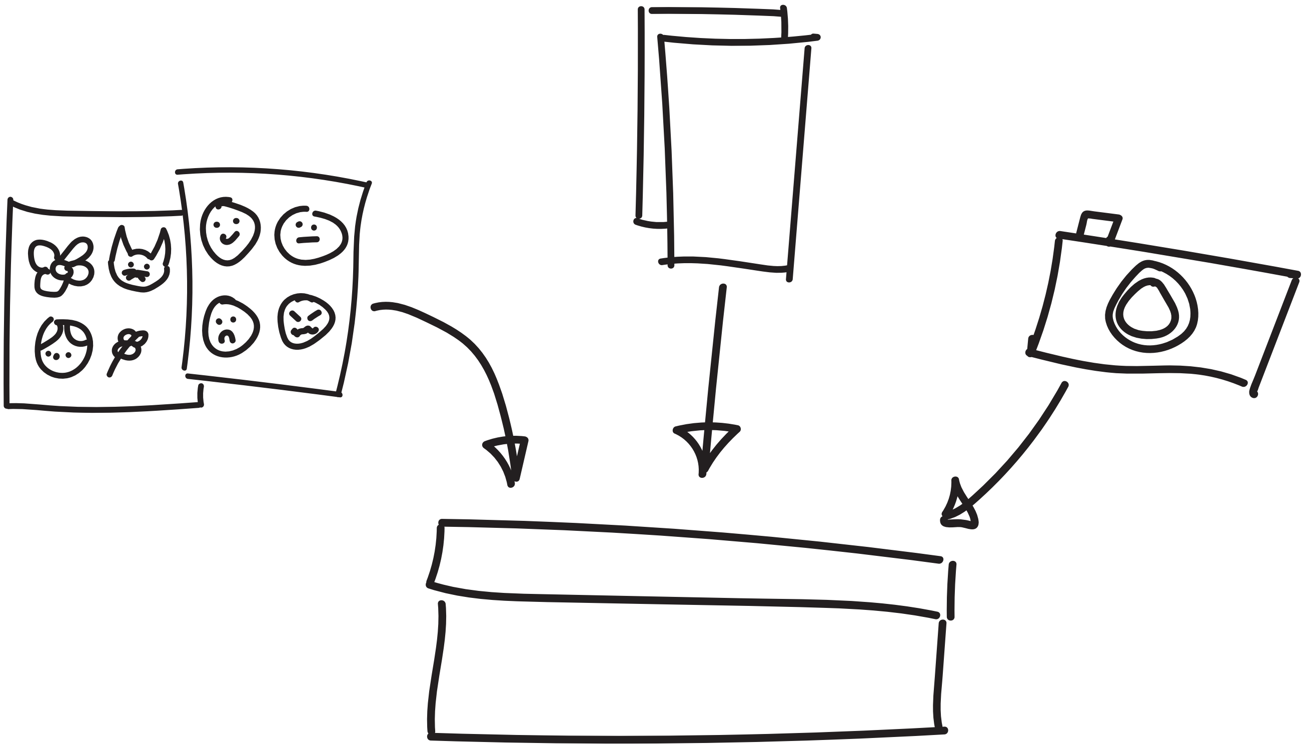

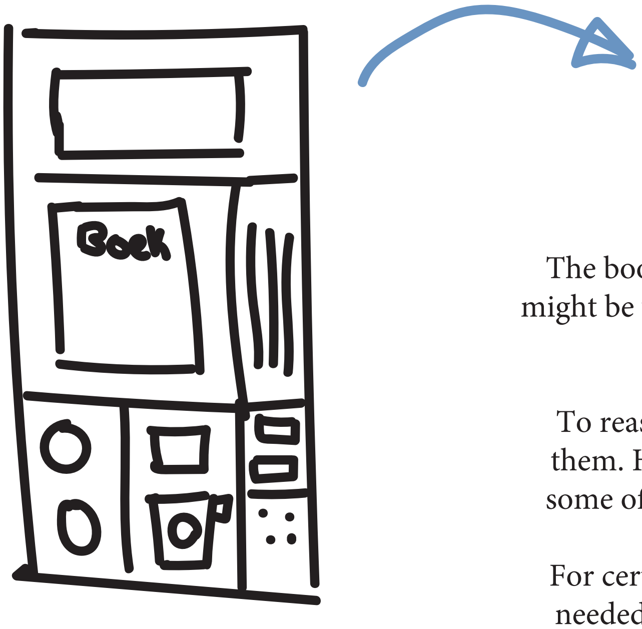

Bag of creativity

The booklet you are currently reading came in a bag. You might be wondering what to do with all the creative materials and the Polaroid camera.

To reassure you right away: there is no obligation to use them. However, if you feel drawn to it, you can complete some of the exercises in the booklet using these materials.

For certain activities, for example, the Polaroid camera is needed - that's why there are photos in the bag. You can think of this box as a place to collect and keep your story.

You may also include objects or items that are important to you, even if they are not specifically requested, and bring them to your sessions. The creative materials can support you in expressing your experiences, thoughts, and feelings visually.

1.

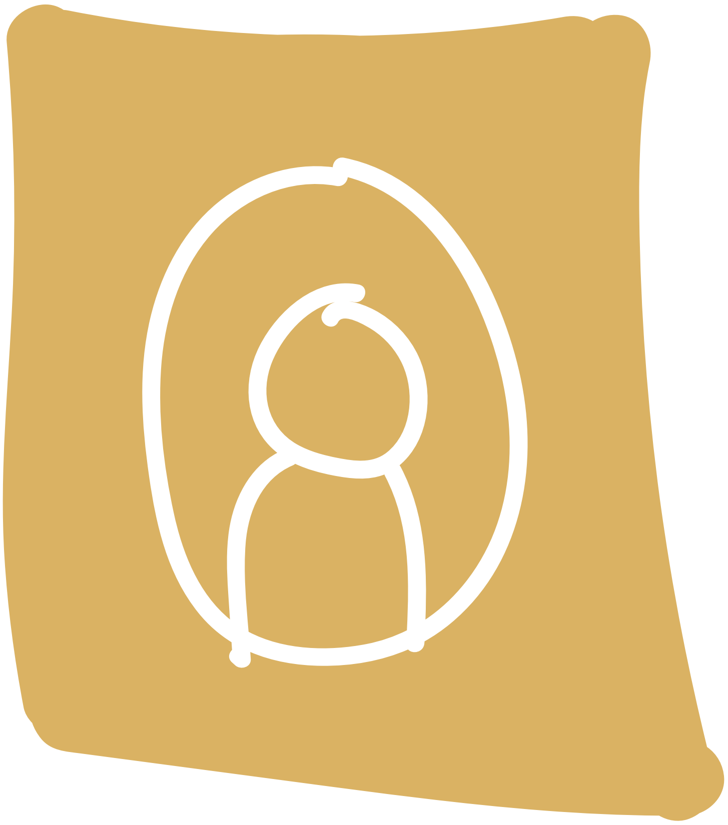

Who are *you*?

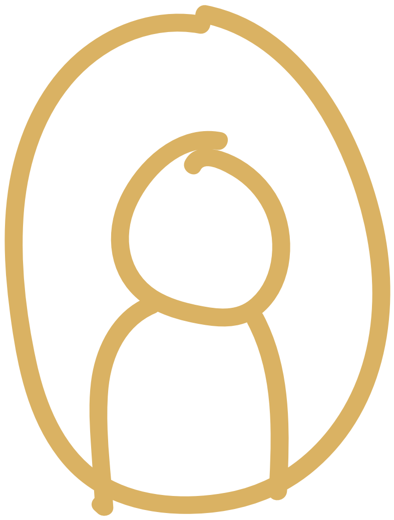

Excercise explanation

Who you are - that's something you probably already know well. However, by reflecting on your hobbies, talents, vulnerabilities, and other topics, you might discover new things that are important to you. That is the goal of this exercise.

On these pages, you can create a profile of yourself that you feel gives a complete picture of who you are as a person. We have highlighted several topics, such as hobbies, energy drainers, energy boosters, talents, and vulnerabilities.

Feel free to also describe or illustrate other topics that are important to you but haven't been asked about yet, using the blank pages provided.

If you wish, you can create a portrait of yourself below or take a selfie with your Polaroid camera

Hobbies

Name

Who are you?

Energy boosters

What do you enjoy doing? What could someone wake you up for, even in your sleep?

Energy drainers

What really annoys you?

Talents & skills

Vulnerabilities

The future

Do you have plans for the future? Where would you like to be? This can be about your health, your work, or even your dreams. Below, you can fill in how you envision your future.

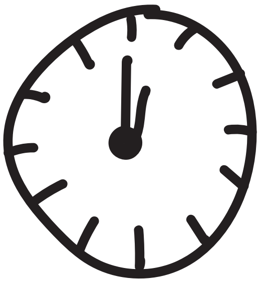

Over half a year:

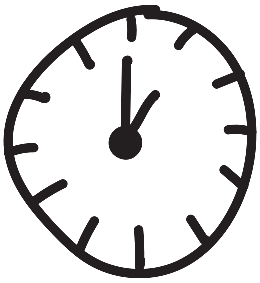

Over one year:

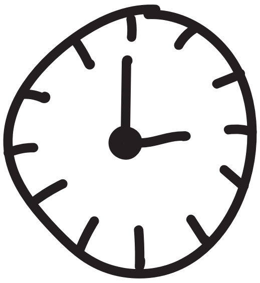

Over two years:

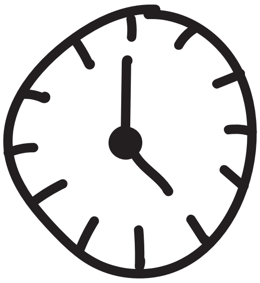

Over five years:

Free space

Below you have space to share anything you would like about yourself. You may write, draw, or express it in any other way you prefer.

Free space

Free space

2.

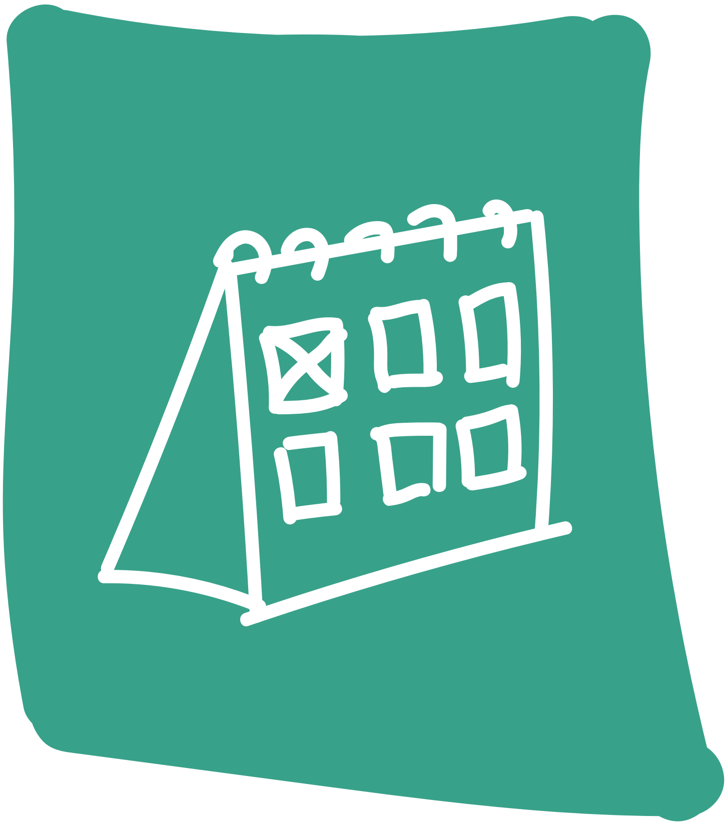

Daily life

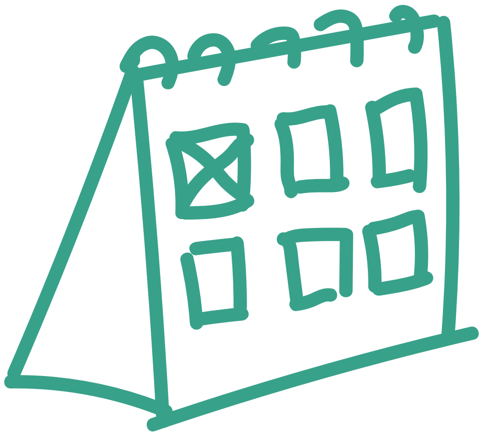

Exercise explanation

What does your daily life look like?
Think, for example, about what and when you eat and drink, your sleep patterns and needs, as well as any education you might be pursuing or work you do.

On the following pages, you can fill in your current situation and your desired situation. Perhaps you are already quite satisfied with how you function now; even then, it can be valuable to complete the exercises.

Are you feeling ashamed of your eating habits, sleep cycle, or whether or not you have work or studies? That is not necessary! If so, you might consider using the third page to write about something in your daily life that you feel less ashamed of and would like to share.

Completing these exercises can provide new insights but, again, they are not mandatory.

2.1

Nutrition

Keep track of what you eat for one day and try the exercise below.

Breakfast:

____

____

Snack:

____

____

Lunch:

____

____

Snack:

____

____

Diner:

____

____

Does your eating pattern look similar on other days?

☐ Yes ☐ No

If no, what is different?

____

____

Are you satisfied with what you eat and drink?

____

____

2.2

Sleep

Keep track of your sleep for one day and try the exercise below.

What time did you go to bed?

What time did you wake up?

Does your sleeping pattern look similar on other days?

- [ ] Yes
- [ ] No

If no, what is different?

Are you satisfied with your sleeping habits?

2.3

Education/work

Do you study, work, or have another daily activity? If you like you can try the exercise below.

What do you do for work, study, or other daily activities?

In the morning:

In the afternoon:

In the evening:

Are you satisfied with your daily activities?

☐ Yes ☐ No

If no, why not?

Blank page

Please write below anything you couldn't share on the previous pages.

3.

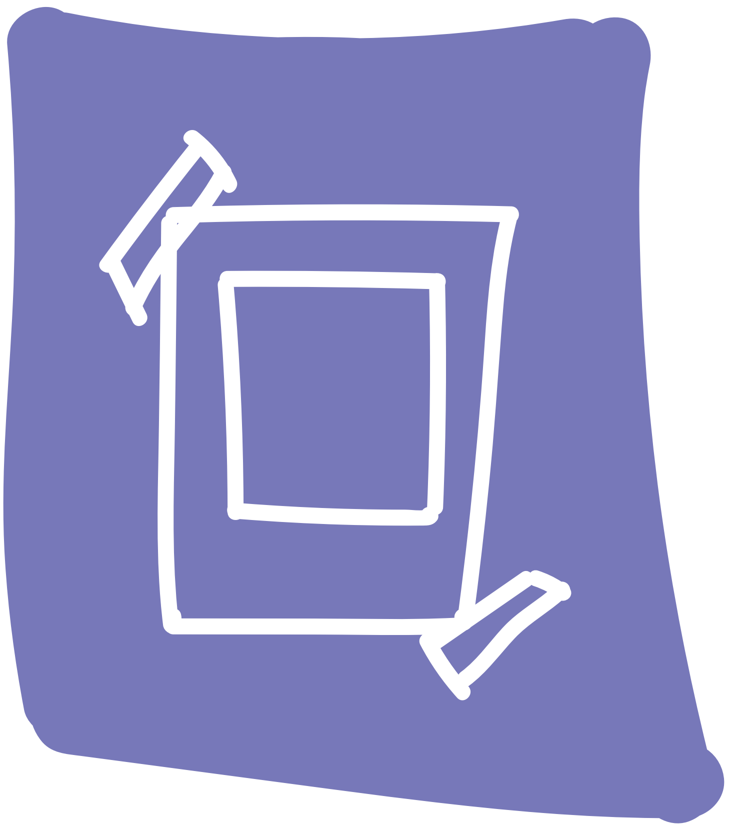

Your **polaroid** experiences

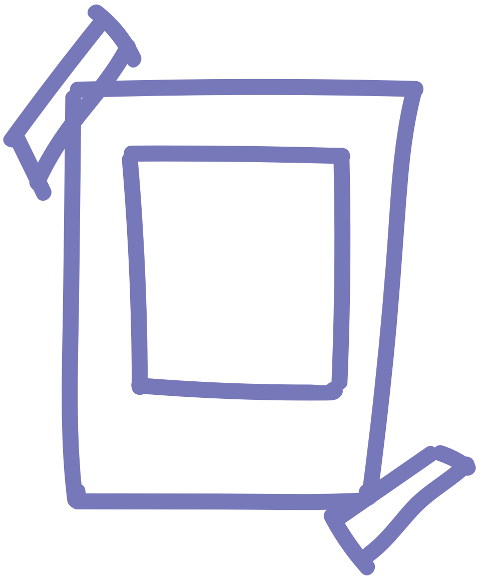

Exercise explanation

Your Polaroid experiences - there are so many words, but sometimes they don't quite capture what you want to say. That's why it can help to represent experiences visually.

In the bag of creativity, you'll find a Polaroid camera. You can use this camera to take pictures of important places, objects, and memories.

Photos can sometimes provide unique insights and can also help others enter your world. Think about places that feel good to you, as well as places you find unpleasant. Write below why, but only if you wish to - nothing is mandatory.

3.1

Your pleasant place(s)

If you like, you can take photos of the place(s) you go to feel comfortable. Paste them into the book and describe why this is a special place for you.

Example

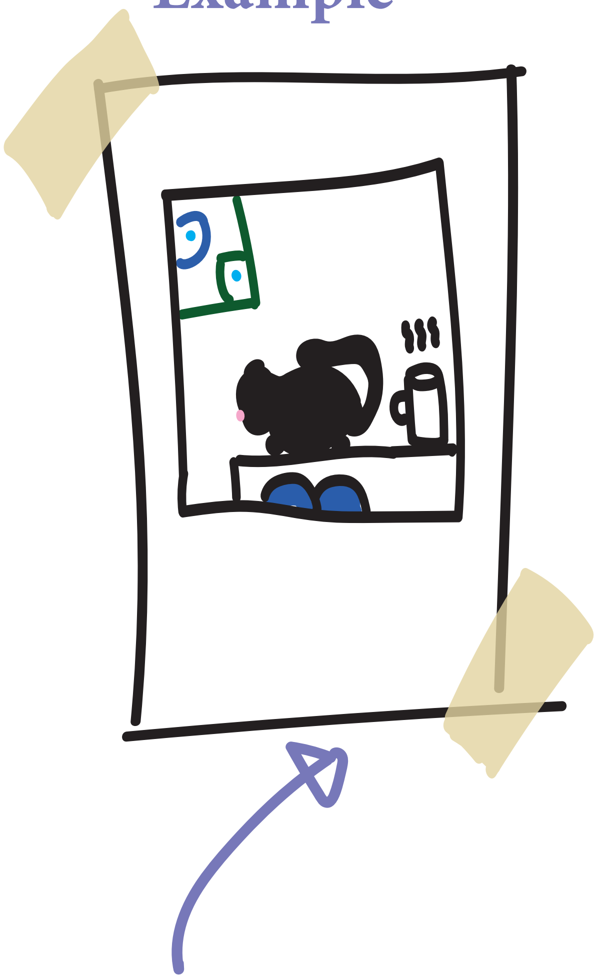

"I got a lovely cat that helps me relax"

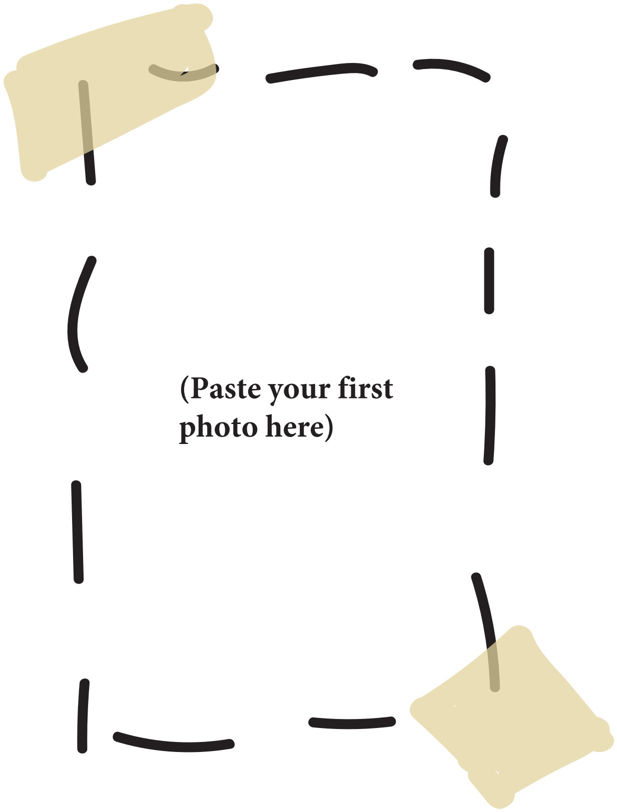

(Paste your
second photo
here)

(Paste your
third photo
here)

3.2

Unpleasant place(s)

If you want, take photos of places you find unpleasant or that trigger negative thoughts and feelings. Paste them into the book and describe why these places are uncomfortable or triggering for you.

Example

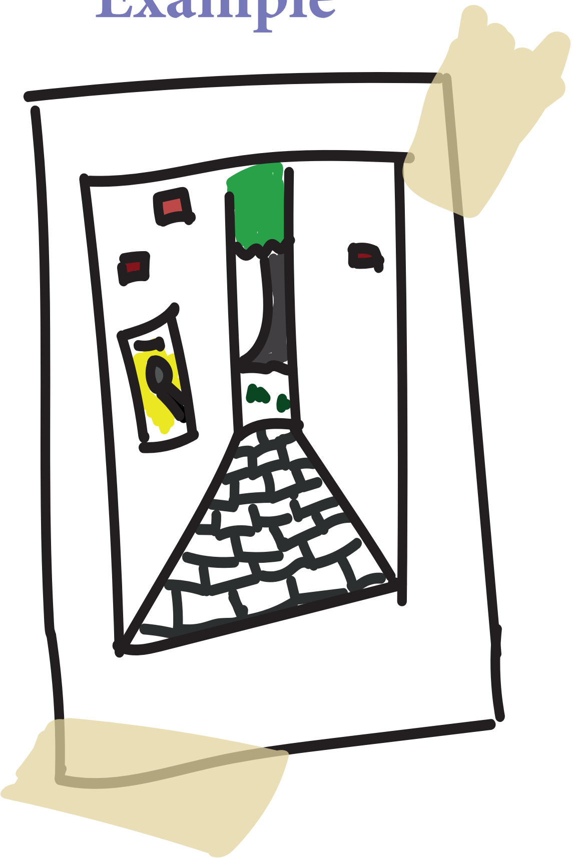

"Alleyways feel very narrow; I prefer to avoid them."

(Paste your first photo here)

(Paste your
second photo
here)

(Paste your
third photo
here)

3.3

Extra photo's

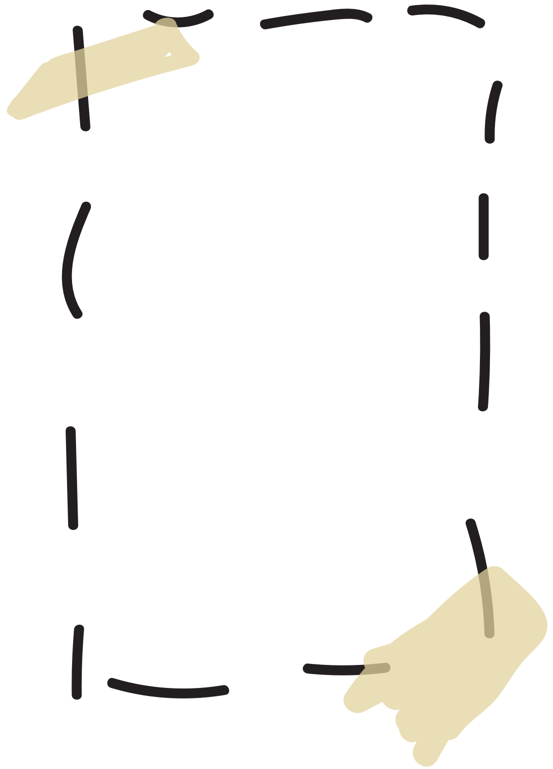

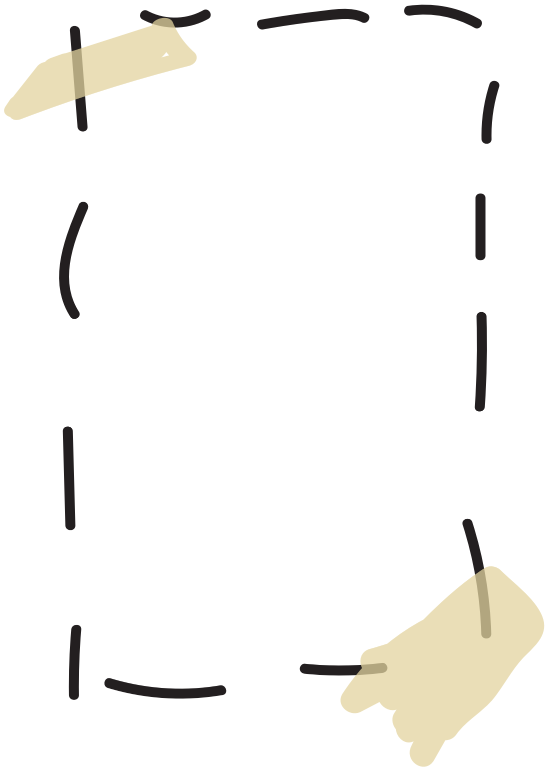

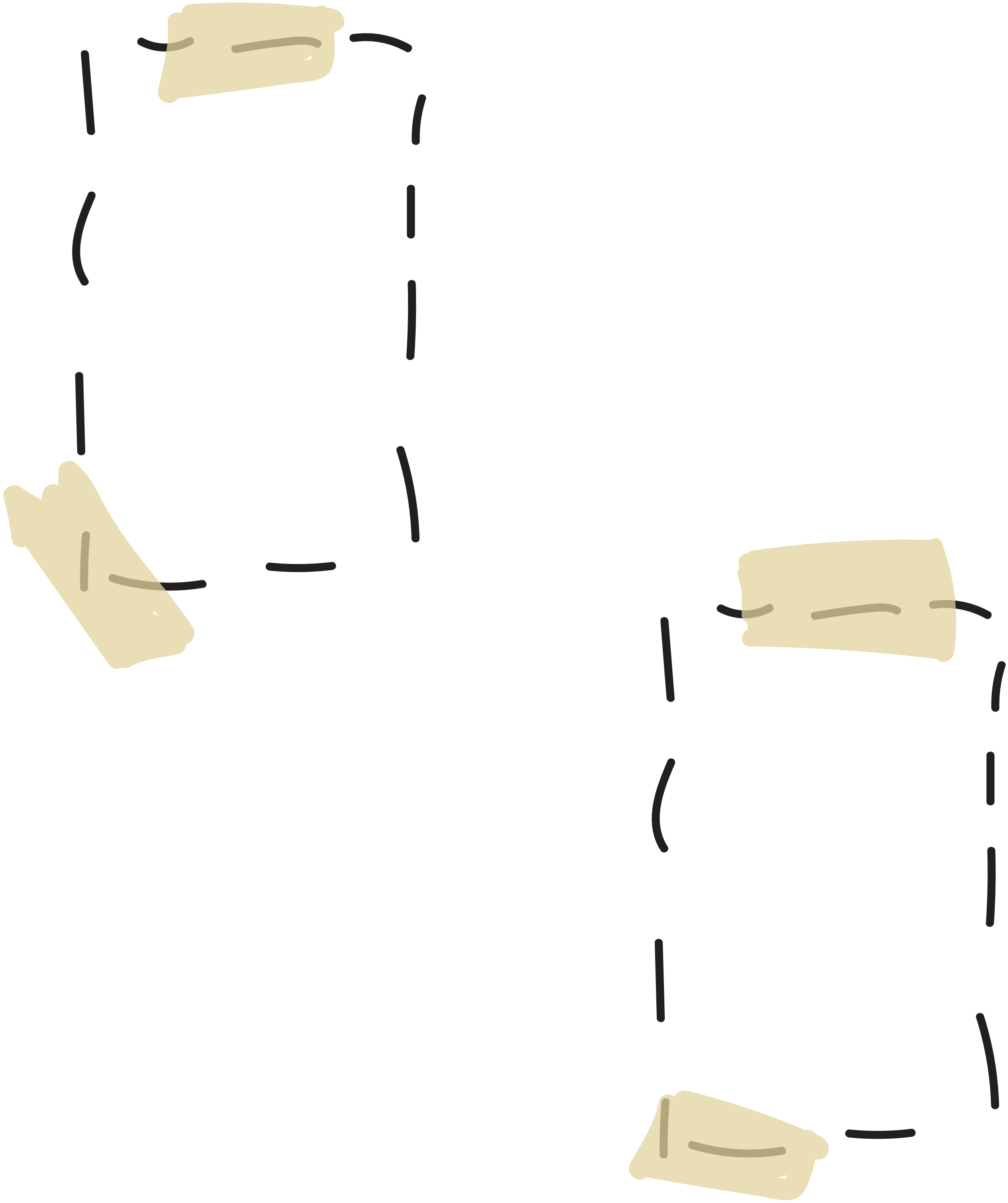

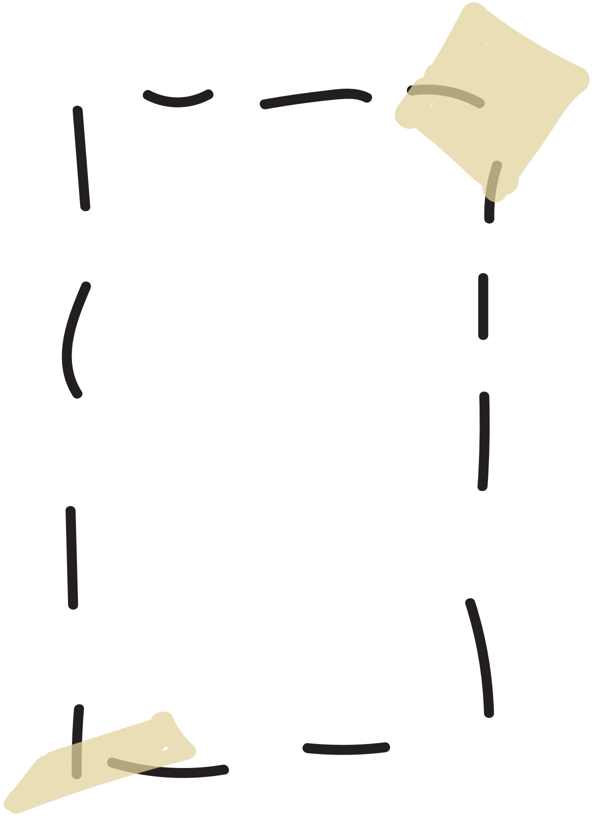

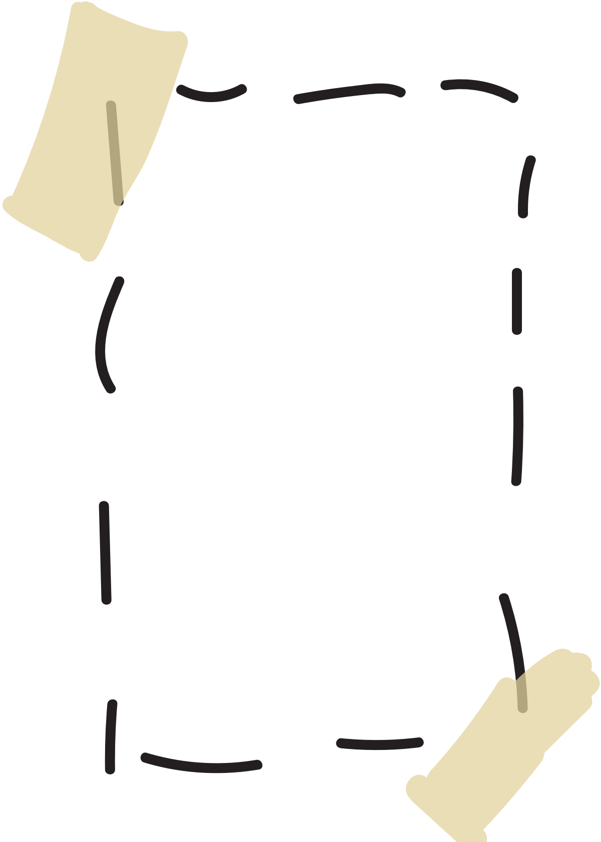

4.

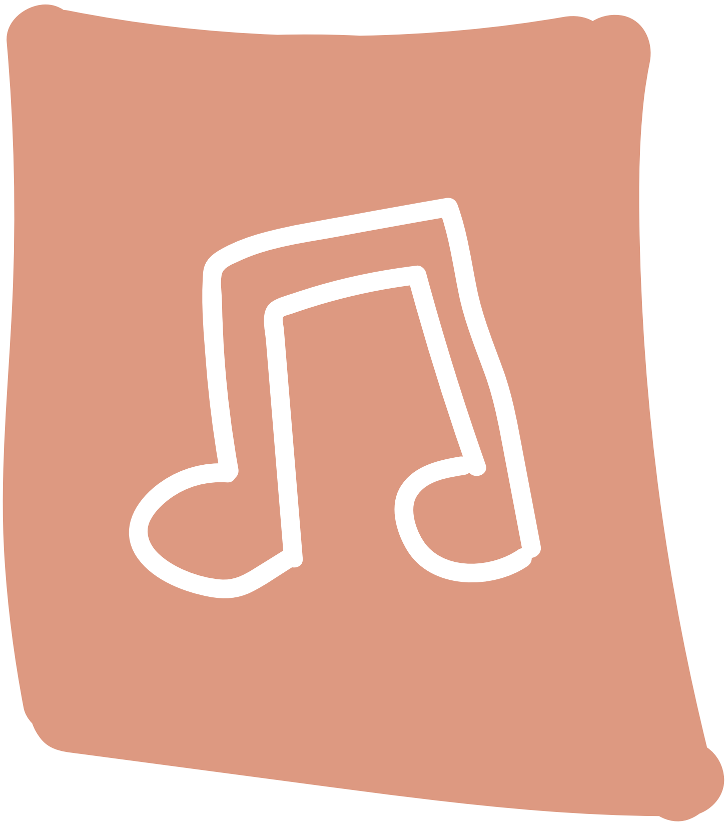

Your playlist

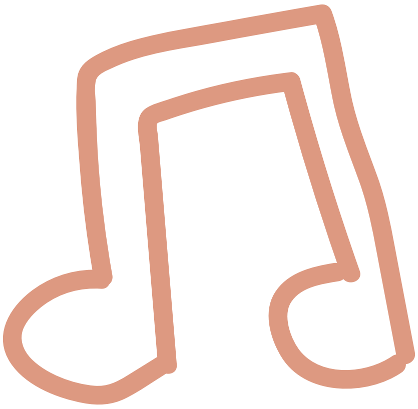

Exercise explanation

Music is very important to many people. It can make you want to dance or help you relax. Music can also touch you on a deeper level - for example, because you relate to the lyrics or because it brings back important memories or gives meaning to your experiences.

On the next page, if you like, you can create a playlist of five songs that mean a lot to you. On the following pages, you can describe what makes these songs so special to you.

4.

Your playlist

Write down the songs you have chosen below. On the following page, also explain what these songs mean to you.

1.

2.

3.

4.

5.

1.

2.

3.

4.

5.

5.

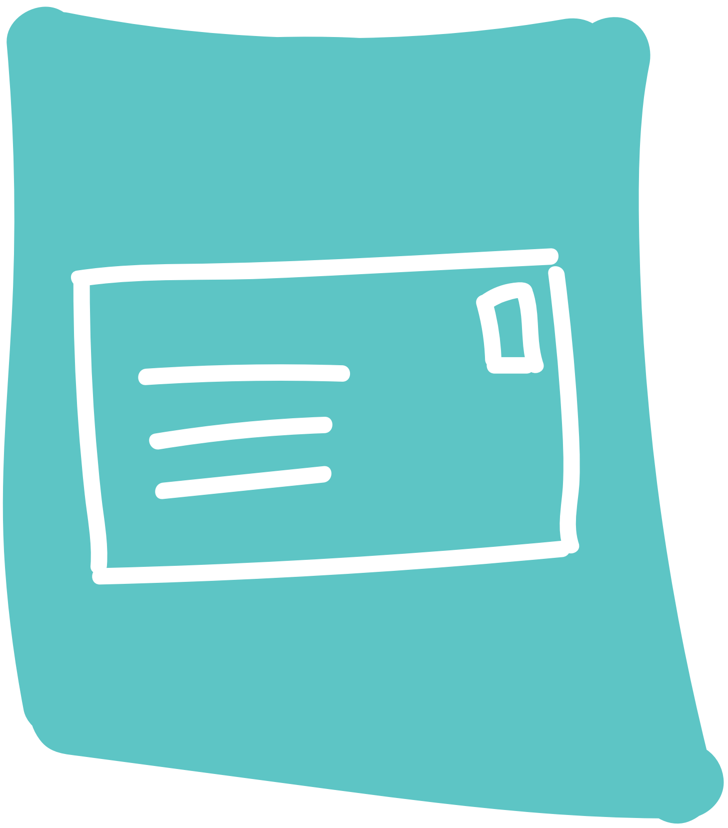

A letter to yourself

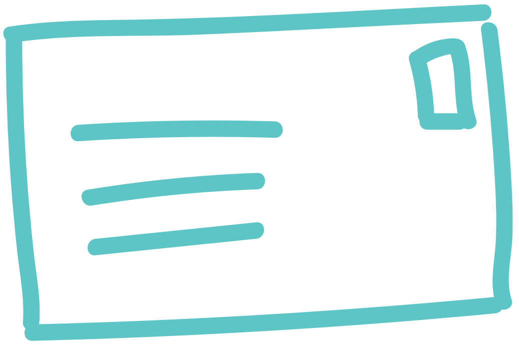

Exercise explanation

You have various wishes and needs. Sometimes it can be difficult to put these into words, but perhaps the previous exercises have already helped you to visualize where you want to go.

On the next page, you can write a letter to yourself about what you wish or hope for in the coming period. This can relate to everything.

You can decorate the front of the page by drawing, adding stickers, or anything you like. On the back, you can briefly describe what you wish or hope for yourself. The stamp is already placed!

5.

Example letter

Front

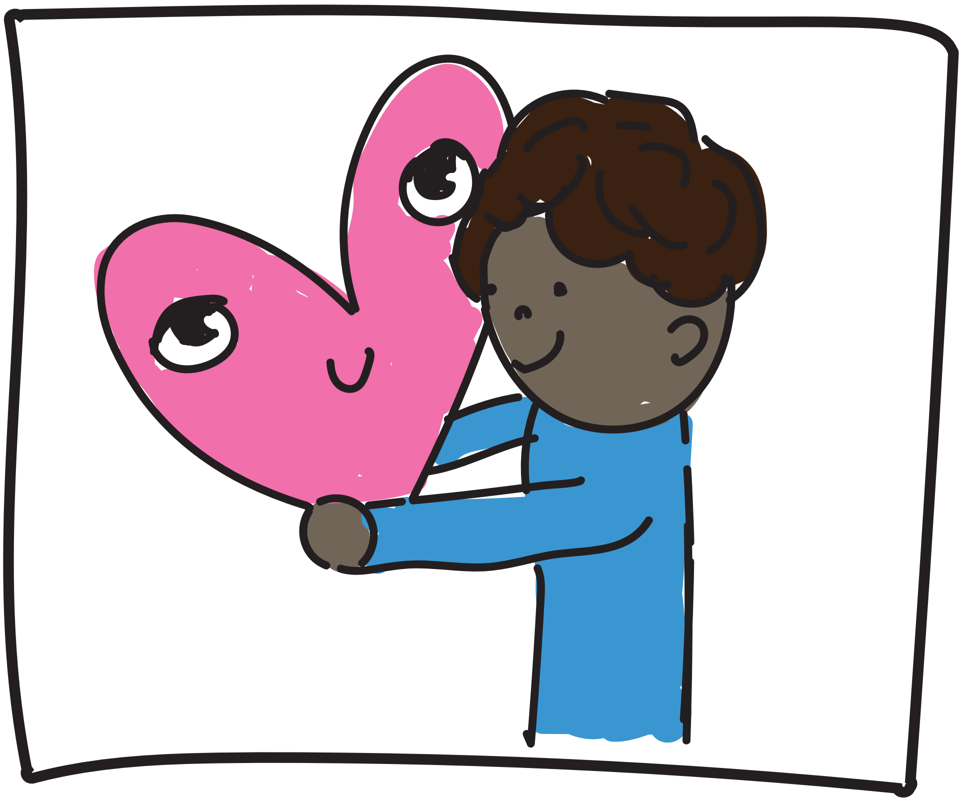

Back

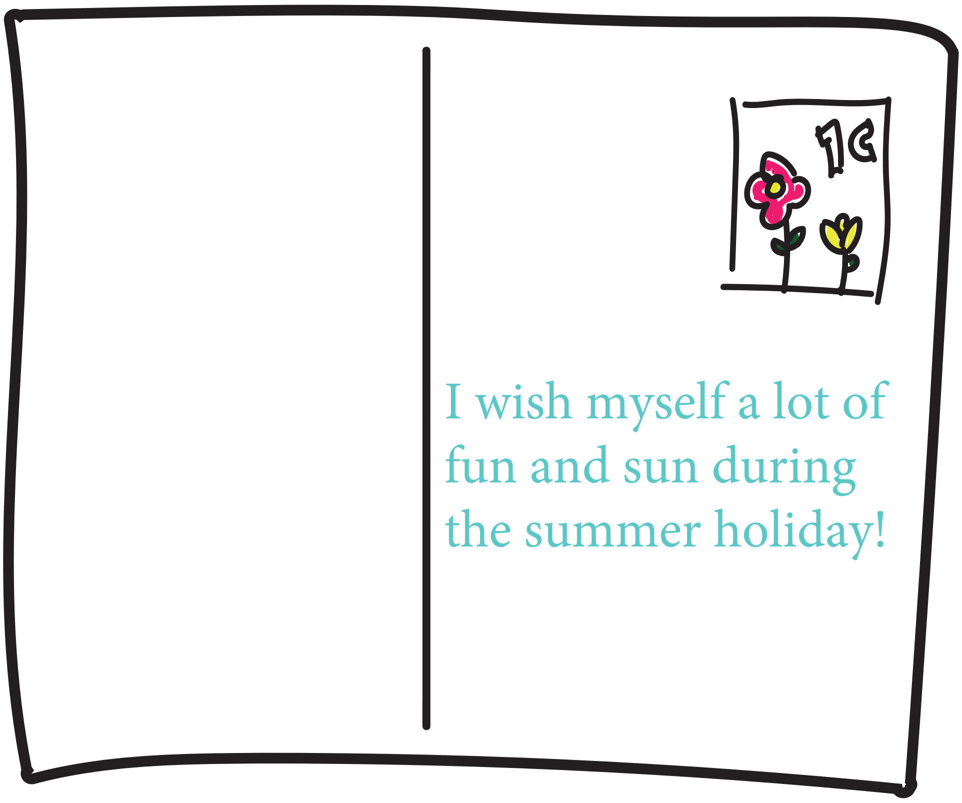

5.

Example letter 2

Front

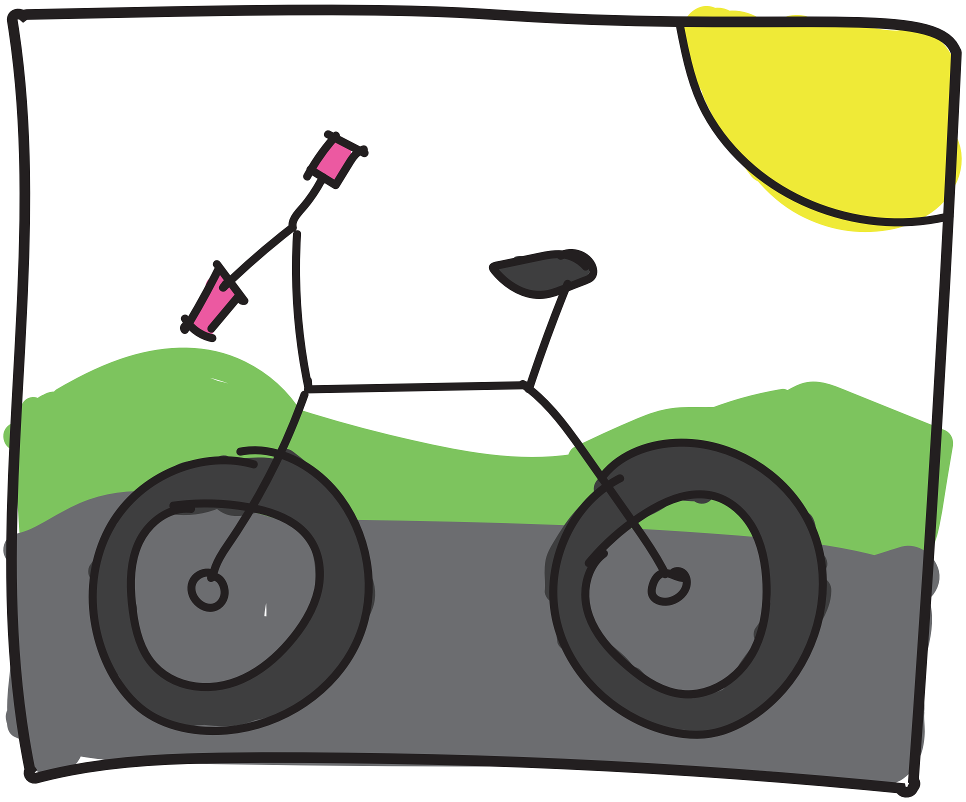

Back

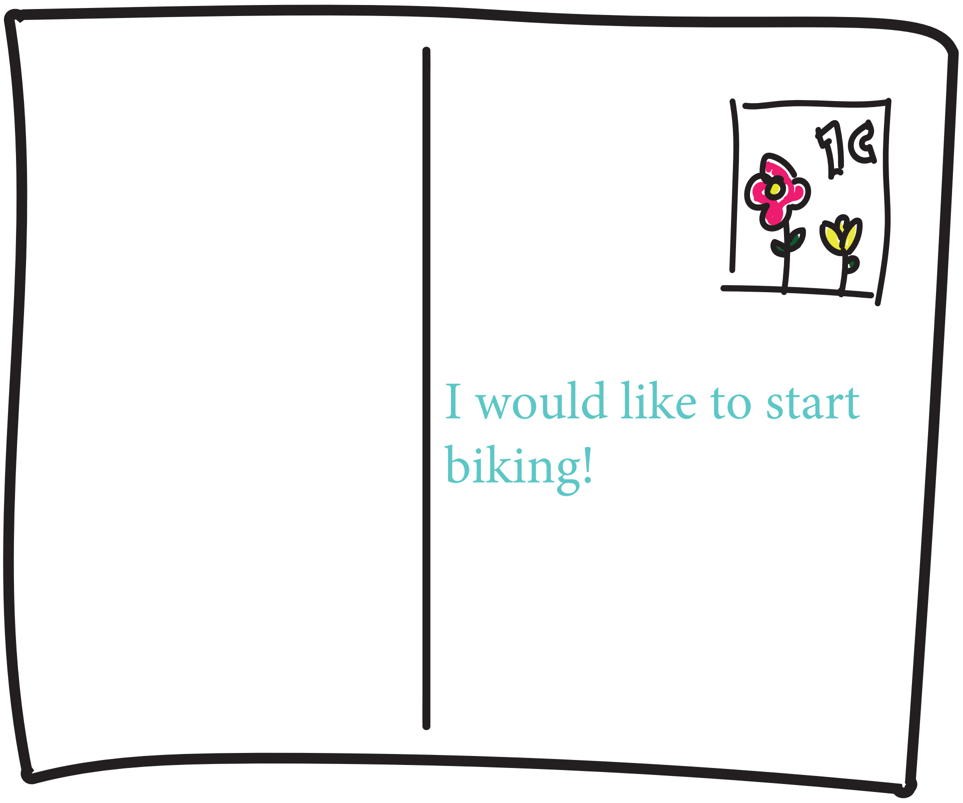

Front

Back

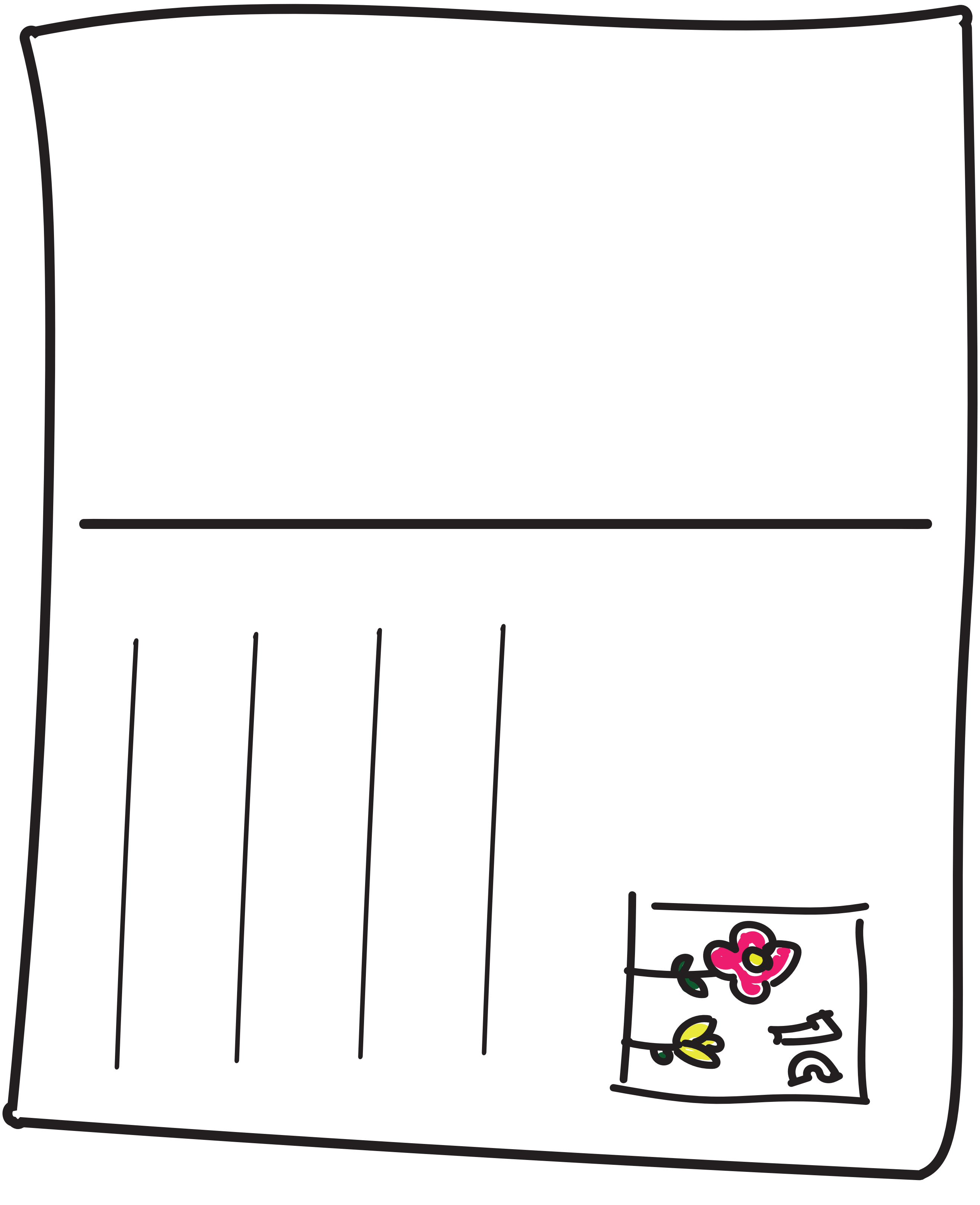

6.

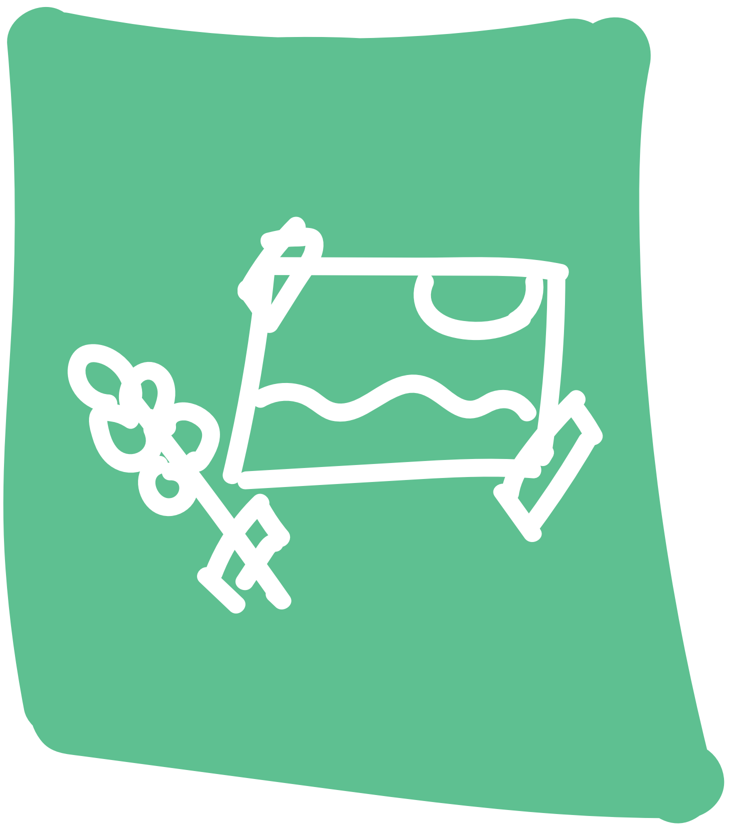

Hobby scrapbook

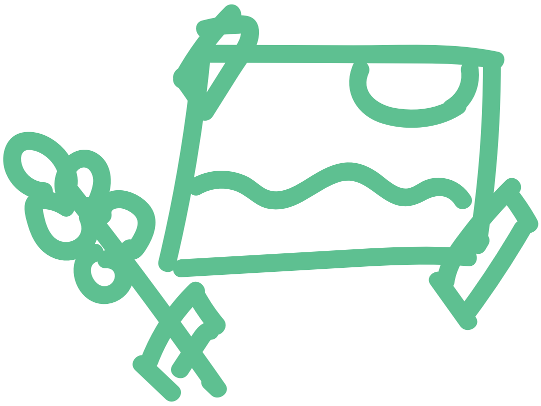

Exercise explanation

What do you enjoy doing in your spare time? What are you good at? And what would you like to do more of? In this hobby scrapbook, you can share everything about your hobbies.

You can paste Polaroid photos of your hobbies here, but also think about newspaper clippings, craft materials - anything that gives a picture of what you like to do in your free time or would like to do more of.

6.

7.

Comic page

Exercise explanation

In the comic panels, you can illustrate something you've been through or memories - whether positive or negative. You can create three different comics: one about the past, one about the present, and one about the future.

On the next page, we've included an example of a comic about the past. You can create your comic using markers or colored pencils, but also with stickers or other materials.

There are no rules, of course. This is your story and your booklet, so you're in charge. Don't be afraid of doing it 'wrong'- it's not about whether you can draw well or not. It's about expressing what's going on in your life in a way that goes beyond just words.

7.

Comic example

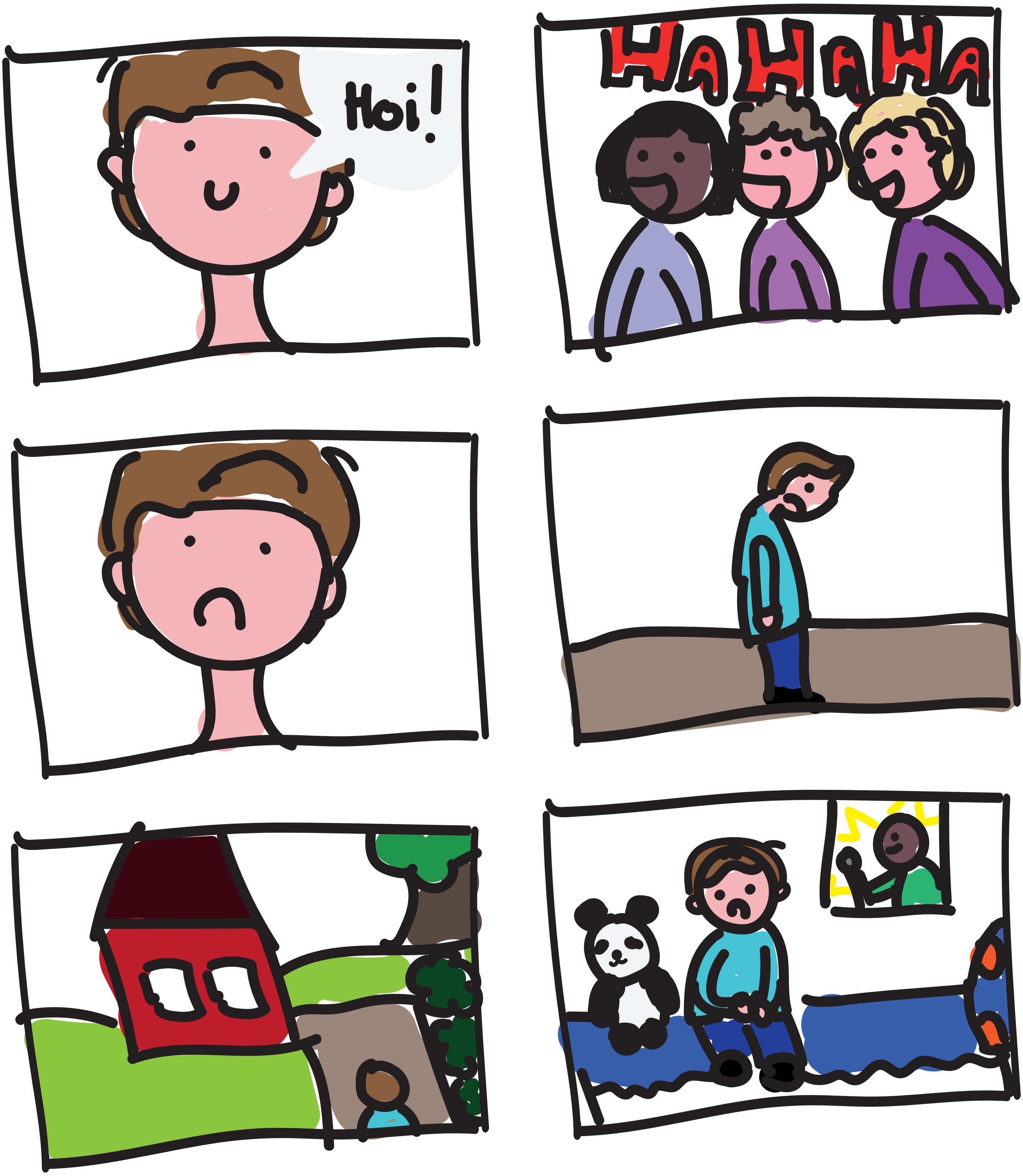

7.1

Comic the past

7.2

Comic the present

7.3

Comic the future

8.

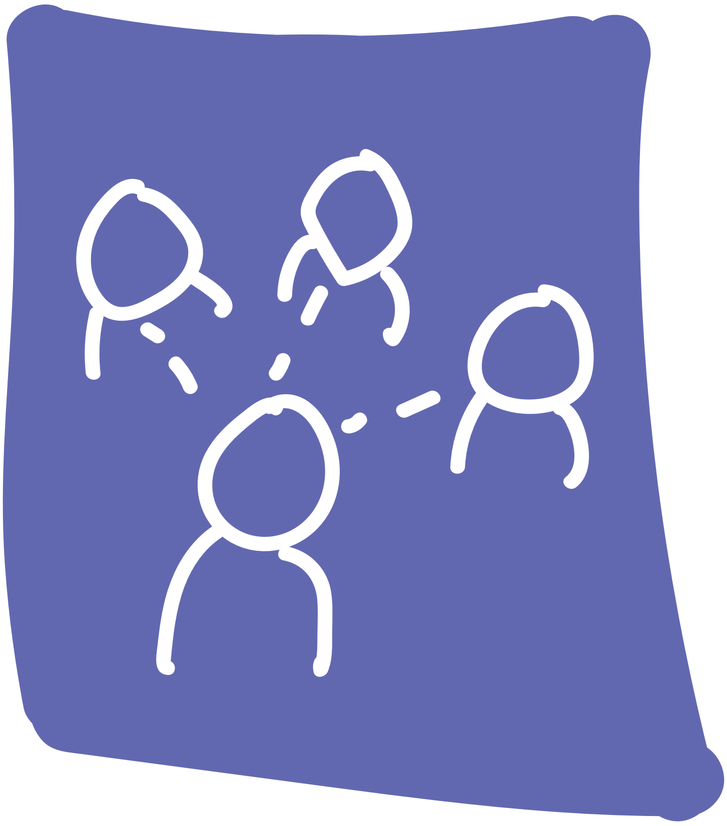

Your social network

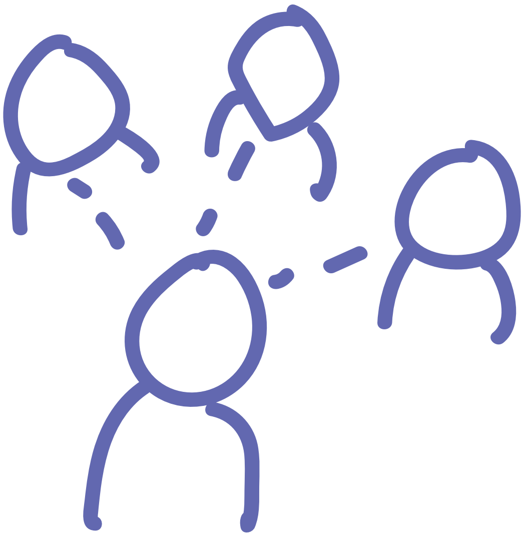

Exercise explanation

Who are the important people in your life, and who do you know? And who supports you - and who might be holding you back?

You can place these relationships on the canvas on the next page.

Think about family, friends, acquaintances, or colleagues. Then, sort them into the helpful and unhelpful categories below.

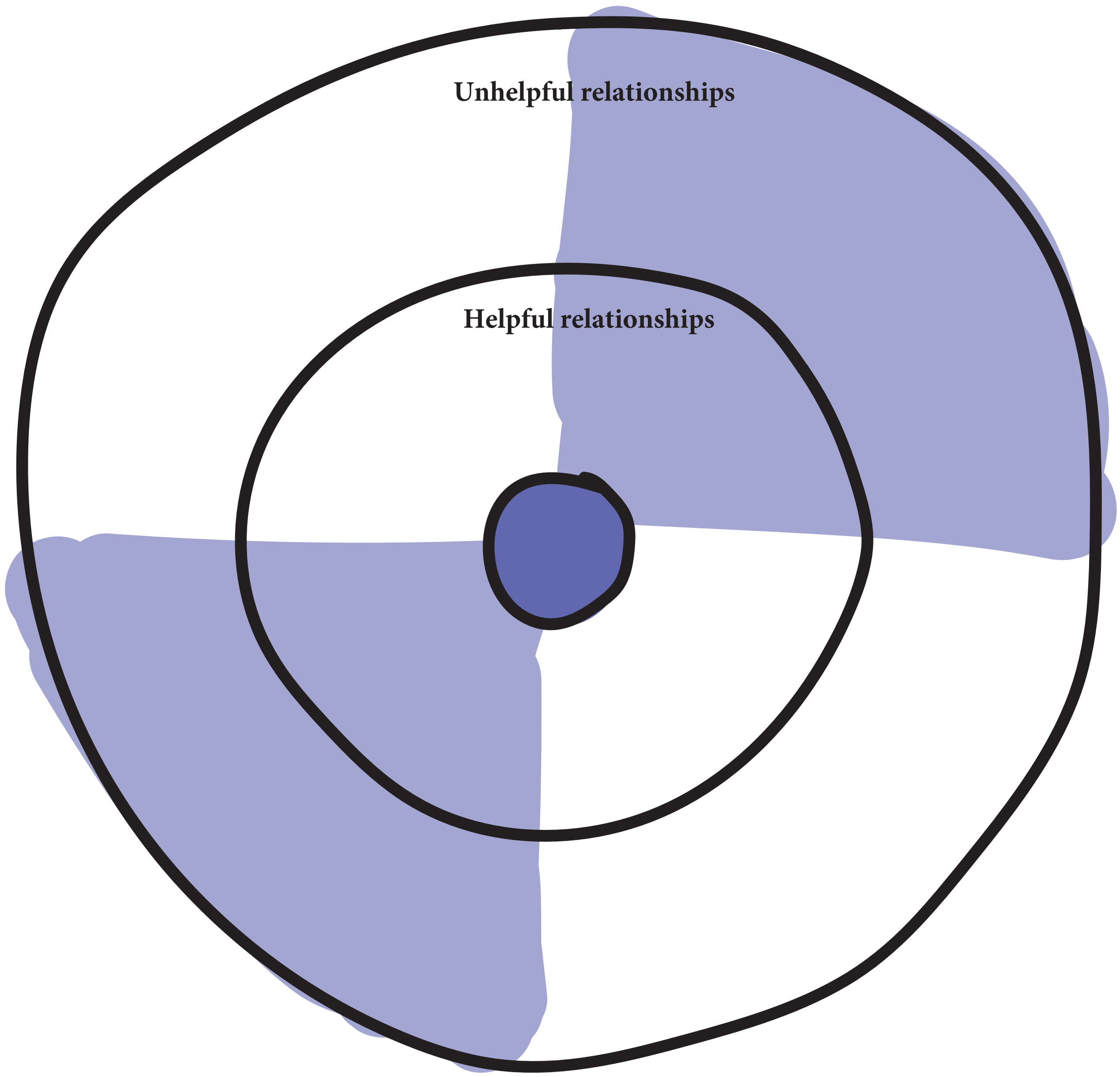

Helpful relationships

Unhelpful relationships

This document was developed in co-creation with people with lived experience, mental health professionals, and researchers as part of the doctoral research project of Lars Veldmeijer titled Design for Mental Health.

This research was conducted in collaboration with NHL Stenden University of Applied Sciences, FAITH-Research, KieN-VIP, UMC Utrecht, and Stichting GGZ-VS.

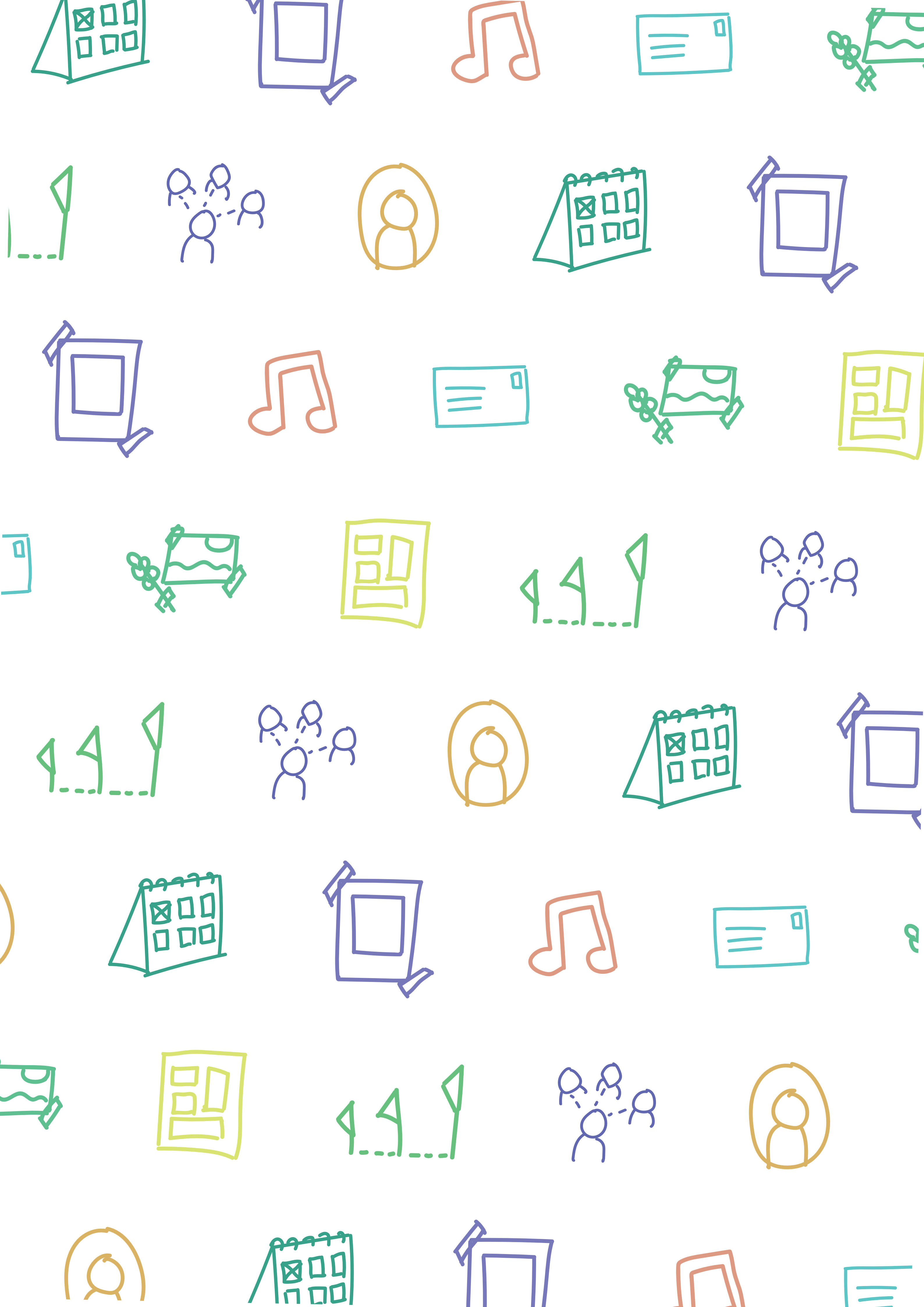

Supplement: Multimedia Appendix 1 [file jopm_v17i1e80184_app1.pdf]
